# Hospital referrals, exclusions from hospital care, and deaths among long-term care residents in the Community of Madrid during the March–April 2020 COVID-19 epidemic period: a multivariate time series analysis

**DOI:** 10.1186/s12877-024-05254-0

**Published:** 2024-08-14

**Authors:** François Béland, Maria Victoria Zunzunegui, Fernando J. García López, Francisco Pozo-Rodriguez

**Affiliations:** 1 Département de Gestion, d’évaluation et de politique de santé, École de santé publique, Université de Montréal, CP 6128 Succursale Centre-Ville, Montréal, QC H3C 3J7 Canada; 2 Institut Lady Davis, Hôpital Juif de Montréal, Montréal, Canada; 3 National Epidemiology Centre, Instituto de Salud Carlos III, Madrid, Spain; 4 Centro de Investigación Biomédica en Red, Enfermedades Neurodegenerativas, Madrid, Spain; 5 Independant Researcher, Madrid, Spain; 6 Département de Médecine sociale et préventive, École de santé publique, Université de Montréal, CP6128 Succursale Centre-Ville, H3C 3J7 Montréal, Canada

**Keywords:** COVID-19, Madrid, Hospital referrals, Long-Term Care Facilities, Resident deaths, Time series

## Abstract

**Background:**

From March 7 to April 7, 2020, the Community of Madrid (CoM), Spain, issued interventions in response to the COVID-19 epidemic, including hospital referral triage protocols for long-term care facility (LTCF) residents (March 18–25). Those with moderate to severe physical disability and cognitive impairment were excluded from hospital referral. This research assesses changes in the association between daily hospital referrals and the deaths of LTCF residents attributable to the triage protocols.

**Methods:**

Daily hospital referrals and all-cause mortality from January to June 2020 among LTCF residents and the CoM population aged 65 + were obtained. Significant changes in LTCF resident daily hospital referrals time series, and in-LTCF and in-hospital daily deaths, were examined with tests for breaks and regimes in time series. Multivariate time series analyses were conducted to test changes in the associations between LTCF resident hospital referrals with daily deaths in-hospital and in-LTCF, and in the CoM population aged 65 + when the triage protocols were implemented.

**Results:**

Among LTCF residents, hospital referrals declined sharply from March 6 to March 23, 2020. Increases in LTCF residents' daily deaths occurred from March 7 to April 1, followed by a decrease reaching pre-epidemic levels after April 28. The daily ratio of in-hospital deaths to in-LTCF deaths reached its lowest values from March 9 to April 19, 2020. The four versions of the triage protocol, published from March 18 to March 25 had no impact on further changes in the association of hospital referrals with daily deaths of LTCF residents in-hospital or in-LTCF.

**Conclusions:**

While LTCF residents’ deaths increased, hospital referrals of LTCF residents decreased with the introduction of the CoM governmental interventions on March 7. They were implemented before the enactment of the triage protocols, protecting hospitals from collapse while overlooking the need for standards of care within LTCFs. The CoM triage protocols sanctioned the existing restrictions on hospital referrals of LTCF residents.

**Supplementary Information:**

The online version contains supplementary material available at 10.1186/s12877-024-05254-0.

## Introduction

Decreases in hospital-based emergency care and planned hospital admissions were observed across numerous high-income countries during the COVID-19 pandemic [1]. Hospitalizations of COVID-19 inpatients in ICU (Intensive Care Unit) and non-ICU (Non-Intensive Care Unit) beds more than compensated for the decrease in non-COVID-19 hospital bed occupancy [1]. Low to moderate levels of hospital bed saturation were experienced in some of these countries [2]. The pressures originating from COVID-19 on the healthcare system in Spain were at least seven times higher on the Verelst et al. [3] pressure intensity scale than those in France, Switzerland, the UK, Belgium, Denmark, Luxembourg, and Sweden. Spain experienced the highest level of COVID-19 mortality among persons aged 65 + among twelve OECD countries [4]. Spain also had the highest long-term care facility (LTCF) resident mortality [4–6],

The *Comunidad Autonoma de Madrid* (Autonomous Community of Madrid – CoM) had the highest excess mortality of all autonomous communities (ACs) in Spain in the Feb25/Apr28-2020 first wave of the COVID-19 epidemic [5, 6]. Nineteen percent of the older adults living in LTCFs in the CoM died [7, 8]. COVID-19 spread in LTCFs as in other countries [9–11]. In a study of data from the USA and 12 European countries, Aaolto et al. found a positive correlation between COVID-19 cases and deaths in LTCFs and the total population [5]. In a study of data from Catalonia [12], the cumulative incidence of COVID-19 mortality in the population was associated with mortality in LTCFs. Mortality in the CoM LTCFs COVID-19 patients was the highest amongst all published studies in Spain. They had more than double the proportion of deaths occurring in LTCFs, compared with studies conducted in regions other than the CoM [13]. Among the policy decisions that may have contributed to these statistics, Koleva et al. [9] identified the protocols restricting hospital referrals of LTCF residents based on triage by public hospital geriatricians issued between March 18 and March 25, 2020 [14–17]

These triage protocols restricted hospital referrals of LTCF residents with moderate to high levels of disabilities and cognitive impairments [13, 14]. Their main goal, allegedly based on evidence, was to ensure coordination between LTCFs and hospital healthcare for LTCF residents with COVID-19. One of the secondary objectives was to prevent the collapse of the CoM healthcare system, specifically of the community’s hospitals. In effect, non-elective hospitalizations increased by 70% from March 14 to March 24, 2020 – from approximately 1000 hospitalizations per day to approximately 1700. Most were COVID-19-related cases [18]. In this context, decreases in hospital referrals of LTCF residents aimed at reducing pressure on ICU and non-ICU bed occupancy.

Healthcare jurisdictions restricted hospital referrals with similar protocols during the first wave of COVID-19. For example, the Government of Québec (Canada) implemented triage protocols limiting access to hospital admission by LTCF residents. Hospital referrals were allowed for LTCF residents with severe COVID-19 symptoms and those needing ambulatory and in-patient care not available in LTCFs. In a cohort of LTCF residents in Montréal, only 5.3% of LTCF residents with severe COVID-19 symptoms were hospitalized in the first wave of the epidemic—March 24, 2020, to July 9, 2020 [19]. Conversely, in the CoM’s triage protocols, the severity of disability and cognitive conditions, not the patients’ COVID-19 condition, restricted access to hospital referrals.

In Spain, Autonomous Communities have the responsibility and decision-making power for policies, funding, organization, and standards concerning LTCFs [20, 21]. LTCFs in the CoM are homes for residents that deliver personal, gerontological, and rehabilitation, but not medical, care. They are responsible for referring residents to healthcare providers as mandated by the Spanish National Health System. The AC's responsibilities were confirmed on March 14, 2020, by the Spanish Government’s Royal Decree 463/2020 establishing a state of emergency in response to the epidemic. ACs ultimately extended medical, nursing, and paramedical care to LTCFs. In addition, referrals of LTCF residents in need of care not available in LTCFs to healthcare facilities able to provide appropriate levels of care were required.

The triage protocols were the most politically challenged public health intervention in response to the epidemic in the CoM. Restrictions on hospital referrals of LTCF residents raised concerns regarding access to vital hospital care and the increased number of daily in-LTCF deaths. Thus, this research focuses on the CoM’s disability-based triage protocols implemented between March 18–25, 2020 restricting hospital referrals of LTCF residents [14, 15]. The study’s objective was to examine the association between the enactment dates of the triage protocols and changes in daily hospital referrals and deaths of CoM LTCF residents in-hospital and in-LTCF.

The objective was examined using the three following approaches:

1. Government of the Community of Madrid (GCM) COVID-19 epidemic interventions between Feb25/Apr28-2020 and LTCF residents' daily hospital referrals and in-hospital and in-LTCF deaths were traced in conjunction.
2. Breaks in the distributions of LTCF residents’ daily hospital referrals, and daily in-hospital and in-LTCF deaths attributable to the enactment of the triage protocols were identified using univariate time series analyses.
3. The extent to which LTCF residents' daily hospital referrals and in-hospital and in-LTCF deaths were associated with the March 18–25, 2020 triage protocols was probed using a multivariable data-generating process.

## Methods

### Material

Data on daily hospital referrals and daily deaths by place of death (hospital or LTCF) of LTCF residents, as well as on daily deaths in the population aged 65 + in the CoM, were obtained. Data extends from January 5 to June 27, 2020, thus covering the COVID-19 epidemic's first wave and the pre- and post-first wave periods. For this work, we define the day of the first hospitalization, February 25, 2020, as the beginning of the COVID-19 epidemic period. The end of the period was set on April 28, 2020, when the Government of Spain published the Transition to a New Normality policy [22]. The pre-COVID-19 period occurred in Jan05/Feb24-2020 and the post-COVID period followed on Apr29/Jun27-2020.

The Transparency Portal of the Community of Madrid *(Portal de Transparencia de la Comunidad de Madrid*), a government office, and the National Statistics Institute (Spain) provide public access to information upon request. Table 1 illustrates the data sources, populations, and periods covered in the analyses.

**Table 1:** Sources of hospital referrals and mortality data

**Source**	**Institution**	**Population**	**Variable**	**Period**
**Transparency Office **	Ministry of Social Policy. Government of the Community of Madrid	Older adults living in Long-Term Care Facilities (LTCF) in the Community of Madrid	Number of daily referrals from LTCFs to reference hospitals	January 1st - June 30^th^, 2020
**Death certificates**	National Institute of Statistics of Spain	Population over 65 years of age in the Community of Madrid	Number of daily deaths	January 1st - June 30^th^, 2020
**Death certificates**	National Institute of Statistics of Spain	Older adults living in Long-Term Care Facilities (LTCF) in the Community of Madrid	Number of daily deaths occurring at LTCFs	January 1st - June 30^th^, 2020
**Transparency Office**	Ministry of Social Policy. Government of the Community of Madrid	Older adults living in LTCFs	Number of daily deaths of LTCF residents occurring at hospitals	March 21^th^ - June 30^th^^,^2020

**Fig. 1:**
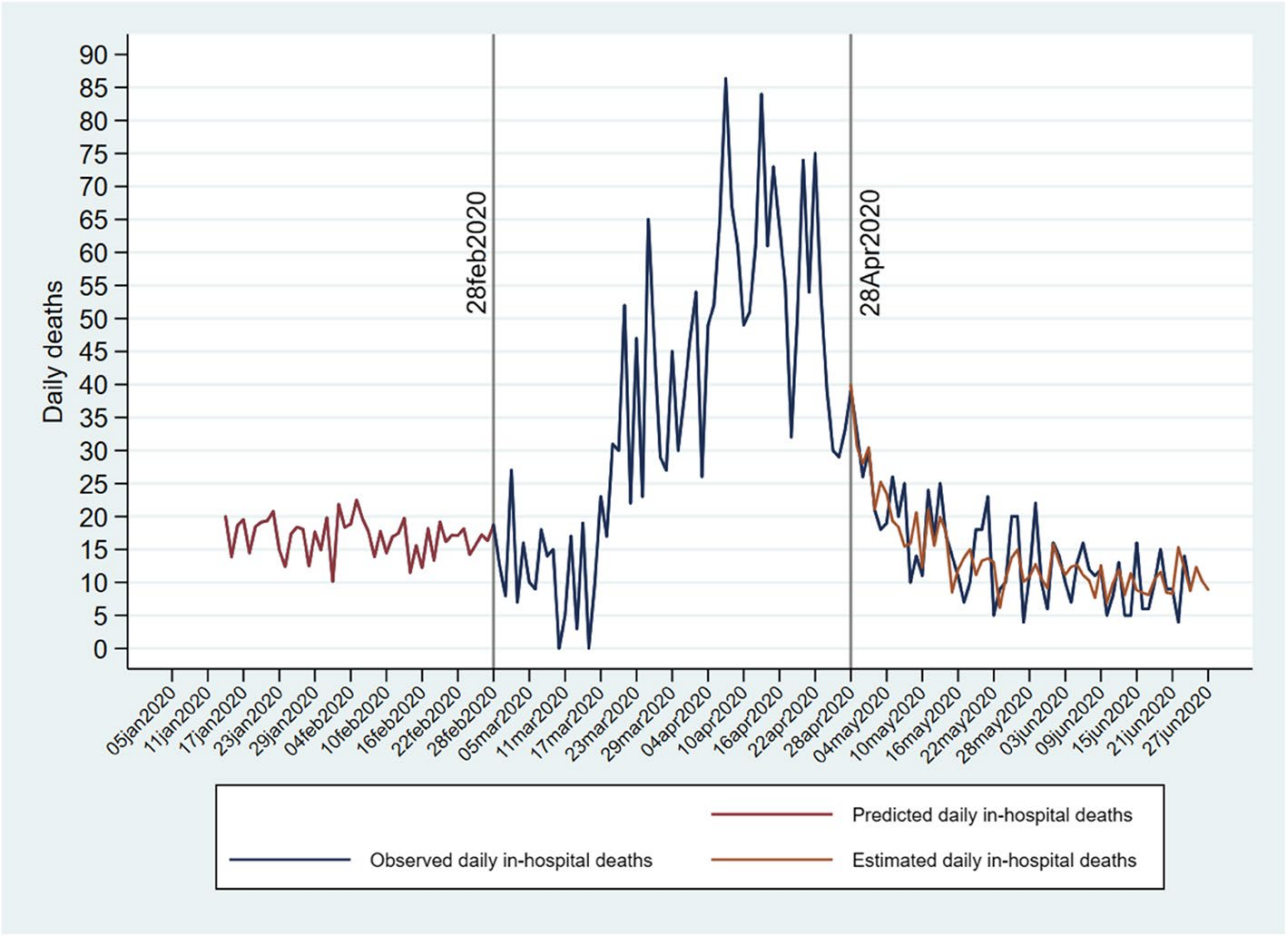
Observed, predicted, and estimated LTCF residents' daily in-hospital deaths

Daily deaths of LTCF residents occurring at hospitals were not available for the pre-COVID-19 period from the National Statistics Institute. They were predicted using a linear regression where predictors were daily deaths of persons aged 65 + living in the CoM, daily deaths in LTCFs, and daily hospital referrals in the COVID and post-COVID period. The results of the regression are plotted in Fig. 1. The pre-COVID predicted values of daily in-hospital deaths are shown in Fig. 1 from January 5 to February 28 (red line). The observed daily in-hospital deaths of LTCF residents are shown by the blue line from February 25 to June 27. LTCF residents’ in-hospital deaths in the post-COVID period were estimated with the regression equation. The observed and estimated daily deaths are plotted from April 28 to June 27. The estimated post-COVID values (green line) follow neatly observed scores as confirmed by the 0.77 correlation coefficient between them. Durbin’s alternative test for autocorrelation rejected the hypothesis of serial correlation among residuals from the regression at the 0.28 P-level. Predicted values of in-hospital deaths of LTCF residents in the pre-COVID-19 period (red line) obtained from the regression were aggregated to the COVID-19 and post-COVID-19 observed daily in-hospital deaths of LTCF residents.

Seven variables were constructed from the data files: 1) Daily deaths in the total population aged 65 years + living in the CoM; 2) Daily deaths in the population 65 + in the CoM living in the community; 3) Total daily deaths of LTCF residents, regardless of the place of death (at LTCF or hospital); 4) Daily in-LTCF deaths of LTCF residents; 5) Daily in-hospital deaths of LTCF residents occurring after hospital referrals; 6) Daily referrals of LTCF residents from LTCFs to hospitals; and 7) Hospital-to-LTCF mortality daily ratios.

### Statistical procedures

#### Data visualization

Graphics were used to represent the observed time series for daily deaths, hospital referrals, and the sequence of administrative interventions, including the March 18–25 triage protocols.

#### Univariate analyses of changes in hospital referrals and daily mortality

The univariate analyses examine whether shocks on daily hospital referrals and deaths were statistically significant and whether they coincided with the date the triage protocols were issued. Shocks were examined using the Ditzen et al. [23] tests. Breaks occur when a time series unexpectedly changes at a point in time. A break may be a one-day shock or can last over some time. In the latter case, the break is followed by a regime lasting until the next break. These statistical analyses (P-level ≤ 0.05) aim to identify the exact day a change occurred in the time series (the breaks) and, if a change lasted more than a day, its starting and ending dates (the regimes).

The operational objective pursued by the triage protocols was to introduce changes – breaks and regimes – in LTCF residents’ hospital referrals. Due to implementation delays, the breaks may have occurred in the days following Mar18-2020, when the first protocol was issued. Hence, the exact break dates and length of the regime for hospital referrals are unknown. The starting and ending dates of statistically significant breaks and regimes in daily in-hospital and in-LTCF deaths during the COVID-19 epidemic are not coterminous with the COVID-19 epidemic starting and ending dates. Thus, they are also unknown. Hence, they were tested with the sequential Ditzen et al. procedure [23] for unknown dates.

Increases in daily variations within a regime may be associated with volatility in a time series [24]. Thus, a high volume of all-cause deaths during the COVID-19 epidemic may have occurred in conjunction with increases in the range of variation of unforeseen patterns of daily deaths during a regime leading to a high level of uncertainty in the flux and management of care to patients in hospitals and LTCFs.

This study used four tests to examine time series attributes [25] and to conclude on stationarity and volatility (see Additional File 1).

### Multivariate analyses of hospital referrals and in-LTCF and in-hospital deaths

**Fig. 2:**
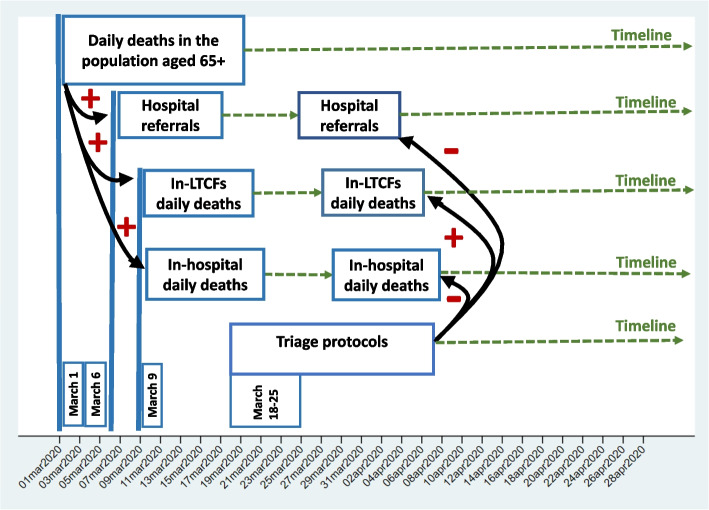
The data-generating process

In this study, the foundational data-generating process was built as a theoretical framework modeling the critical sequences of events during the Feb25/Apr28-2020 COVID-19 epidemic. Two sequences of events were considered as “impulses” generating “responses” in other events. Only the direct responses from the two foundational impulses are considered in the data-generating process (Fig. 2).

The first impulse was the increase in daily deaths in the CoM population aged 65 + . The changes in daily hospital referrals and in-hospital and in-LTCF daily deaths were responses to this impulse (Fig. 2). The contribution of deaths in the population 65 + in the CoM to hospital referrals and in-LTCF and in-hospital deaths of LTCF residents was postulated to be positive before the publication of the triage protocols – see the positive signs in Fig. 2 alongside the arrows. Thus, the number of hospital referrals and LTCF resident in-hospital and in-LTCF deaths were increased by deaths in the population 65 + in the CoM in the ascending phase of the COVID-19 epidemic.

The second impulse consisted of the four triage protocols enacted by the CoM from Mar18/25–2020. The first protocol was issued approximately four weeks after the hospitalization of the first suspected COVID-19 case in the CoM on Feb25-2020. In line with the triage protocols' primary and secondary goals, the following events should have occurred: 1) Hospital referrals decreased after their enactment (negative sign in Fig. 2). 2) Concomitantly, LTCF resident in-hospital deaths decreased (negative sign in Fig. 2). However, 3) with the decrease in LTCF resident in-hospital deaths, an increase in the number of in-LTCF deaths was expected (see the positive sign in Fig. 2).

From Fig. 2, a multivariate model with three equations was designed to obtain estimates of daily hospital referrals and daily deaths of LTCF residents in-LTCF and in-hospital:

Equation 1:

- Response: LTCF residents’ hospital referrals;
- Impulse_1: Daily deaths in the population aged 65+ in the CoM;
- Impulse_2: The triage protocols.

Equation 2:

- Response: Daily in-LTCF deaths;
- Impulse_1: Daily deaths in the population aged 65+ in the CoM living in the community;
- Impulse_2: The triage protocols.
- Impulse_3: LTCF residents' hospital referrals.

Equation 3:

- Response: Daily in-hospital deaths among LTCF residents;
- Impulse_1: Daily deaths in the population aged 65+ in the CoM living in the community;
- Impulse_2: The triage protocols.
- Impulse_3: LTCF residents' hospital referrals.
- Impulse_4: Daily in-LTCF deaths.

In Eq. 1, daily deaths in the population aged 65 + in the CoM include persons in private households and LTCFs. The GCM decision to restrict hospital referrals of LTCF residents was introduced in the ascending phase of the COVID-19 epidemic in the population [16, 17]. In Eqs. 2 and 3, only deaths in the population 65 + in the CoM living in the community were considered as LTCF resident deaths were the responses.

In the multivariate model, implementation of the triage protocols were introduced as breaks and regimes. They were coded into three variables: shifts, pulses, and ramps [23]. 1) Shifts, i.e. sudden and one-day shocks in the time series were coded "1" on the day of the event and 0 otherwise. 2) Pulses, i.e., non-varying shocks over time, were coded "1" on days included in the regime and 0 otherwise. 3) Ramps, i.e., temporary changes, were coded from 1 to T, increasing by one unit for each day of the associated regime until the end “T” of the regime [25].

Stationarity and volatility tests were conducted to guide the choice of the multivariate time-series model [26]. Three time series models were considered as candidates: the vector autoregressive model (VAR), the vector error-correction model (VECM), and the MGARCH model [26].

In time series models, the associations between responses through time are considered through lagged time series coefficients. Thus, the multivariate time series model includes three contributors: 1. Previous responses' daily values – the response lagged coefficients; 2. The contribution of the impulses was examined in two parts: a) The break and regime coefficients for impulses identified in the univariate analyses; and b) The time series coefficients for impulses. Therefore, the larger the contribution of response lagged coefficients, the smaller the contribution of the impulses to the responses. Statistical analyses were run on Stata 15 [27].

## Results

### A narrative of GCM interventions in the Feb25/Apr28-2020 COVID-19 epidemic and of LTCF residents' daily hospital referrals and daily in-LTCF and in-hospital deaths

**Fig. 3:**
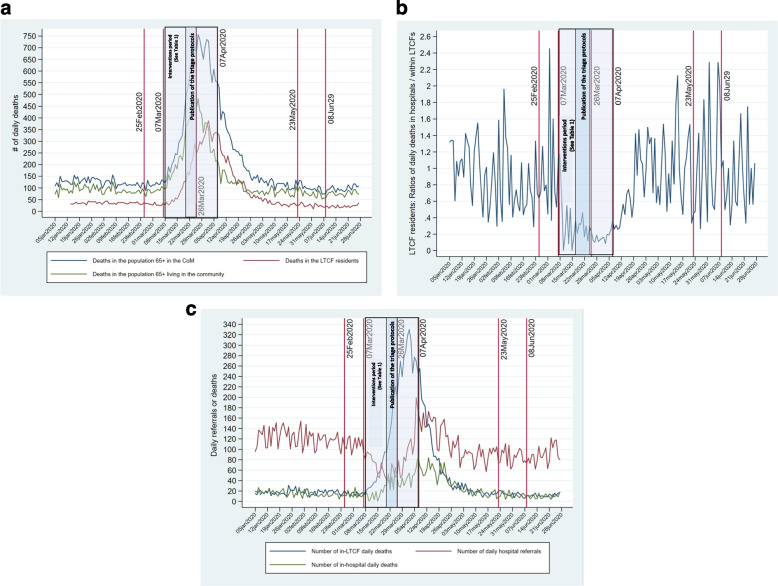
Times series for all-causes daily deaths, hospital referrals, and GCM interventions. **a** Daily deaths in the population 65 + in the CoM and LTCFs. **b** Hospital-to-LTCF ratios of daily deaths of LTCF residents. **c** LTCF residents' daily referrals and in-hospital and in-LTCF daily deaths

The CoM’s Directorate General of Public Health (DGSPCM-*Dirección General de Salud Pública de la Comunidad de Madrid*) issued a procedure for hospitalization of suspected cases of COVID-19 a month before the ascending phase of daily deaths in the population aged 65 + . The ascending phase in LTCF residents began 10 days later (Fig. 3a). The first hospitalization of a suspected case in the CoM occurred on Feb25-2020 [28]. Centralized management of hospital beds was initiated on Mar7-2020 (Table 2). Also, the same day, a three-phase Hospital Resource Elasticity Plan was promoted (see[18] pages 45–50).

**Table 2:** Events in the first wave of COVID-19 in the Community of Madrid

Date of the event	Events
January 30	Reporting of a COVID-19 case to the Public Health Direction of the Community of Madrid (DGSPCM-Direccion General de Salud Publica de la Comunidad de Madrid). The Unit assessed the need for hospitalization using four criteria
February 25	First hospitalization of a suspected case in the CoM
February 27	The first known case of COVID-19 in the Community of Madrid was confirmed
March 3	The first death from COVID-19, a 99-year-old woman living in an LTCF, was confirmed
March 3	Closing Day Social Care Centers and LTCFs was considered a priority by the DGSPCM
March 7	Centralized management of hospital beds initiated
March 8	LTCFs were locked down. Residents were not allowed to leave LTCFs. Visits from family members or friends were banned (see [29] page 42). The DGSP brief also recommended that elderly persons or persons with chronic diseases stay at home or in the LTCF where they live
March 12	According to the *Plan de Accion contra el coronavirus* (Action plan against coronavirus) published by the *Consejeria de Salud de la Comunidad de Madrid* (CSCM—Ministry of Health of the Government of Madrid) LTCF residents were to be treated within LTCFs premises. No evidence of medicalization of LTCFs were found
March 12	LTCF managers informed the *Consejera de Politicas Sociales de la Comunidad de Madrid* (CPSCM – Ministry for Social Policies of the Community of Madrid) that they were experiencing problems with the residents’ hospital admissions
March 17	Local LTCF administration reported that LTCF residents’ referrals were impossible. Many of them died thereof
March 18—25	The first administrative directive restricting the hospitalization of LTCF residents was issued by the CSCM. Residents with moderate disabilities or severe cognitive impairments were excluded from transfers to the hospital. At each hospital, a liaison geriatrician was responsible for the triage of potential patients living in LTCF requesting hospitalization. Consultations with the liaison geriatrician were initiated by the LTCF staff and carried out by phone Four versions of the protocol were issued between March 18 and March 25. Triage criteria were changed with marginal consequences for admission to hospitals. Administrative directives were applied for several weeks and entered gradually into disused The Spanish Geriatricians (SEGG) published an extended protocol for the management of care in LTCFs in times of COVID-19
March 22	The first patients were admitted to the hospital IFEMA, a 1300-bed field hospital. Until its closing on May 1, four thousand patients were admitted at the IFEMA. The March 18–25 administrative directives were enforced in the case of referrals of LTCF residents to IFEMA
March 26	The Government of the Community of Madrid announced in a press release a “Shock Plan” for LTCFs. Changes in the responsibility for the delivery of care were planned. No changes to the exclusion criteria for hospital referrals of LTCF residents were proposed
April 7	The March 18–25 administrative directives restricting access and delivery of hospital care were never withdrawn officially by the GCM. However, the head of the CSCM, Enrique Ruiz Escudero, in a radio interview, mentioned their gradual retreat
April 28	The Spanish government issued the *Plan Para la Transicion Hacia una Nueva Normalidad* (Transition Plan for a renewed normality). Four phases were defined from the state of the COVID-19 epidemic on April 28 to the full normalization expected at the end of June. Indicators for the transition between phases were defined. Regional authorities were not expected to transit at the same pace from one phase to another
May 23	The Community of Madrid was authorized to access Phase 1 on May 23 and Phase 2 on June 8

Restrictions on hospital referrals were issued in the first and third weeks of March 2020. From March 8, the date of the lockdown of LTCFs, the GCM gradually introduced restrictions on access to healthcare outside of LTCFs (Table 2). In addition, there was evidence that LTCF managers encountered increasing barriers to access of their residents to hospitals [28, 30, 31]. As in-hospital deaths of LTCF residents decreased while in-LTCF deaths increased, the ratios of the former to the latter started to decrease abruptly after Mar8-2020 (Fig. 3b), five days after the start of the GCM intervention period and ten days before the publication of the first protocol.

Compared to the pre-COVID-19 period, hospital referrals from LTCFs decreased abruptly in the Mar6/17–2020 period (Fig. 3c; Table 3). According to the GCM Ministry of Health action plan (Mar12-2020), LTCF residents were to be treated on-premises, and the medicalization of LTCFs was announced (Table 2). While the medicalization of LTCFs was never defined by the GCM Ministry of Health, in the 2020 GCM Ministry of Health Annual Report [18], guidelines for the medicalization of hotels included the ability to provide the means to control the spread of COVID-19 among residents and staff, to reduce necessary hospitalizations, and to increase the ability to meet needed care. Organizational and clinical links with hospitals were also planned. Medicalization was defined by the May6-2020, order of the High Court of Justice of Madrid as providing LTCFs with medical and nursing staff, and the appropriate ways and means necessary to care for LTCF residents during an epidemic [30]. However, the GCM Ministry of Health never implemented the medicalization of LTCFs [32].

**Table 3:** Average daily number of deaths and hospital referrals in LTCFs

	**Average daily number of deaths: LTCF residents**					**Hospital **	**Ratios of periods after March 07 to the before March 07 period**			
	**Total **	** in LTCF**		** in hospitals**		**Referrals**	**Total**	**In LTCFs**	**In hospitals**	**Hospital**
		**N**	**%**	**N**	**%**	**N**				**Referrals**
**Before the first death from COVID-19 in a CoM LTCF:**										
** January 05-March 06 **	33	18	54.5%	15	45.5%	121	1.00	1.00	1.00	1.00
**Before the administrative directives:**										
** March 07-March 17**	41	30	73.2%	11	26.8%	86	1.24	1.67	1.34	0.71
**Shortly after the administration directives: **										
** March 18-March 25**	172	136	79.1%	36	20.9%	51	5.21	7.56	1.45	0.42
**Population aged 65+ daily deaths peak:**										
** March 26-April 06**	314	271	86.3%	42	13.4%	93	9.52	15.06	1.58	0.77
**Population daily deaths peak to New Normalization phase 1:**										
** April 07-May 22**	193	131	67.9%	62	32.1%	113	5.85	7.28	1.24	0.93
**After New Normalization phase 1:**										
** May 23-June 27**	33	17	51.5%	16	48.5%	88	1.00	0.94	0.94	0.73

March to May 2020 were tense epidemic times:

1. On Mar18/25–2020, average daily deaths in LTCF increased, reaching a ratio of 5.21 compared to Jan5/Mar7-2020 (Table 4). Also, hospital referrals reached their lowest levels (Fig. 3c; Table 3).
2. The GCM’s “shock plan” (*Plan de choque*) [33] was made public on Mar26-2020 (Table 2) at the beginning of the period (Mar26/Apr6-2020) of the highest levels of daily deaths in the CoM population aged 65 + and in LTCF residents (Fig. 3a; Table 3). The daily in-LTCF death average peaked at 15 times the pre-COVID-19 period daily death average (Table 3), while the daily death average of hospitalized LTCF residents was only 1.58 times higher (Table 3).
3. The gradual retreat of GCM administrative directives restricting access and delivery of hospital care began on Apr7-2020 (Table 2; Figs. 3a, b, c) when the ratios of daily deaths in the CoM population aged 65 + and in LTCF residents decreased (Table 4).
4. After the New Normalization Phase 1 implementation on May23-2020 (Table 2), these ratios deepened below unity, except for the total deaths of LTCF residents (Table 3).
5. Finally, the daily hospital referral average in the pre-COVID-19 period was higher than in the ascending phase of the epidemic and even higher than in the post-COVID-19 period (Fig. 3c; Table 3).

### Univariate time series analyses of changes, vis-à-vis the enactment of the triage protocols, LTCF residents’ daily hospital referrals, and daily in-hospital and in-LTCF deaths

**Fig. 4:**
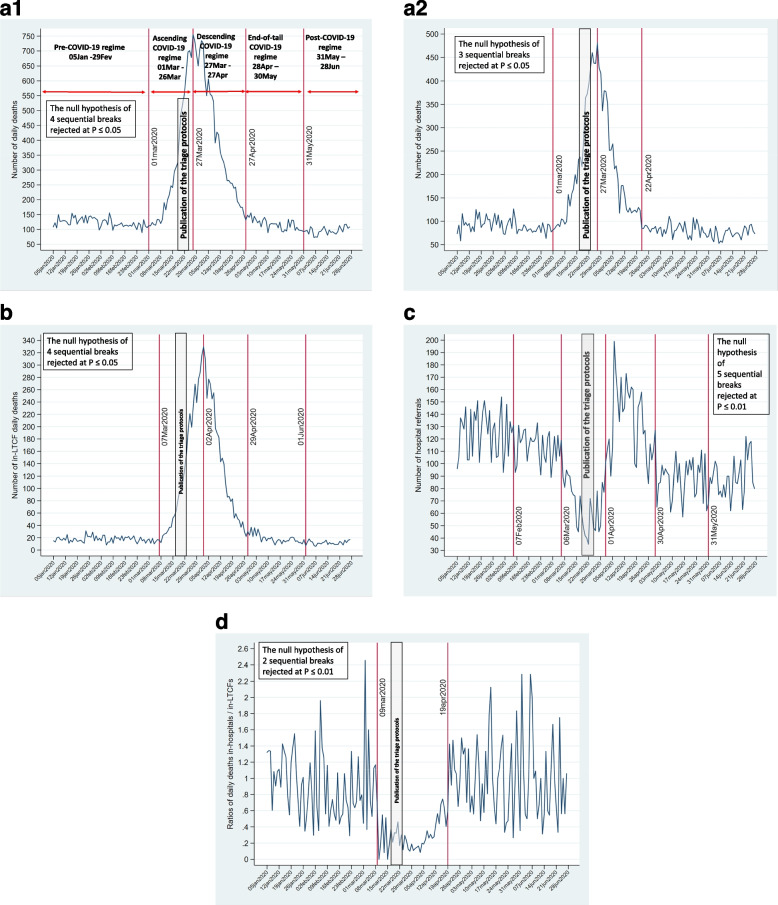
Breaks and regimes in the impulses and responses: **a1** Daily deaths in the population 65 + in the CoM. **a2**. Daily deaths in the population 65 + in the CoM living in the community. **b** Daily in-LTCF deaths. **c** Daily hospital referrals. **d** Ratios of in-hospital/in-LTCF daily deaths: persons living in LTCFs

Changes were modeled as breaks and regimes in the respective first difference time series, and the velocity of changes as volatility. The breaks, the regimes, and the results of the Ditzen et al. [23] procedures are shown in Figs. 4a to 4d for breaks and regimes and S1a to S1d for volatility (See Additional File 1). To enhance the figures' readability breaks are shown on time series, not on the first differences.

Changes in deaths in the population aged 65 + in the CoM introduced by shocks are illustrated by four breaks (Fig. 4a.1: March 1, 2020; March 27, 2020; April 27, 2020; and May 31, 2020). Five regimes were identified: pre-COVID-19; ascending and descending phases of the epidemic; end-of-tail COVID-19; and post-COVID-19. The first two breaks in the population 65 + living in the community happened on the same date as the break for the total population (Fig. 4a.2). The first two breaks in the population aged 65 + in the CoM were followed by about a week by the breaks in in-LTCF deaths (Fig. 4b). No statistically significant break was associated with in-hospital daily deaths (Figure not shown). Thus, daily hospital deaths did not exhibit statistically significant abrupt changes.

**Table 4:** Average daily number of deaths in the population 65+ in the CoM

	**Average daily number of deaths**		**% of deaths**	**Ratios on pre-COVID-19 periods **	
	**Population aged 65+ **	**LTCF residents**	**LTCF on**		
			**population 65+**	**Population aged 65+ **	**LTCF residents**
**Before the first death from COVID-19 in a CoM LTCF:**					
** January 05-March 06 **	123	33	26.8%	1.00	1.00
**Before the administrative directives:**					
** March 07-March 17**	187	41	21.9%	1.52	1.24
**Shortly after the administration directives: **					
** March 18-March 25**	540	172	31.9%	4.39	5.21
**Population aged 65+ daily deaths peak:**					
** March 26-April 06**	666	314	47.1%	5.41	9.52
**Population daily deaths peak to New Normalization phase 1:**					
** April 07-May 22**	335	193	57.6%	2.72	5.85
**After New Normalization phase 1:**					
** May 23-June 27**	112	33	29.5%	0.91	1.00

Breaks and regimes in hospital referrals are shown in Fig. 4c. Hospital referrals fell to their lowest point in the Mar6/31–2020 regime. The triage protocol publication period was included in this regime (Fig. 4c). It occurred in the ascending phase of the Feb25/Apr28-2020 COVID-19 epidemic in the CoM and the LTCFs (Figs. 4a, b). No break in hospital referrals was associated with the Mar18/25–2020 triage protocols.

Two breaks were significant in the time series for the ratios of deaths in-hospital / in-LTCF (Fig. 4d). The regime was defined by the breaks that started on March 9, five days after the closing of LTCFs (Table 2), and ended 11 days after the Apr7-2020 gradual retreat of the GCM administrative directives restricting access and delivery of hospital care for LTCF residents (Table 2). The regime in the hospital-to-LTCF ratios of daily deaths coincided with the regime of intervention of the GCM. The Mar18/25–2020 triage protocols were encompassed within that period.

### Multivariate time-series analyses of the association of LTCF residents' daily hospital referrals and daily in-hospital and in-LTCF deaths with the March 18–25, 2020 triage protocols, using a multivariable data-generating process

**Fig. 5:**
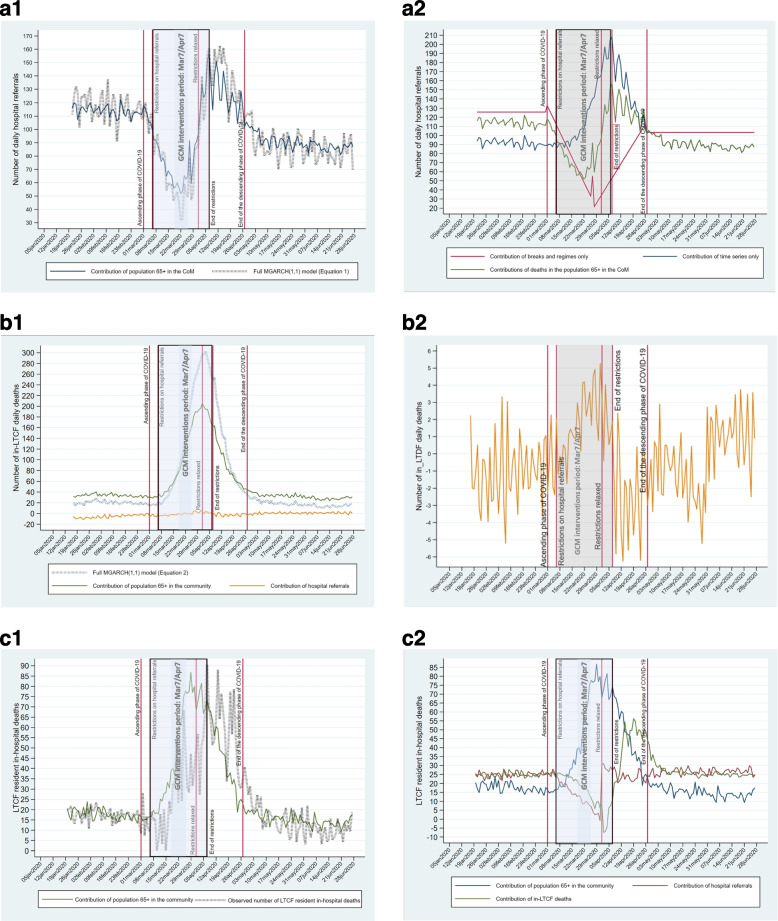
Contribution of impulses to responses in the three MGARCH equations (Table S2). **a1**. Contribution to hospital referrals of the time series for deaths in the population 65 + in the CoM. **a2**. Contribution to hospital referrals of the time series, breaks, and regimes for deaths in the population 65 + in the CoM. **b1**. Contribution to in-LTCF deaths of the time series for deaths in the population 65 + living in the community and hospital referrals. **b2**. Contribution to in-LTCF deaths of the time series for hospital referrals. **c1**. Contribution to LTCF resident in-hospital deaths of the time series for deaths in the population 65 + living in the community. **c2**. Contribution to in-hospital deaths of time series, breaks, regimes for referrals, community and in-LTCF deaths

The results of the MGARCH(1,1) model are available in Table S2 Additional File 2. The fits of the full MGARCH(1,1) model to the observed response time series were high (Additional File 2, Figures S2.1a, b, c) with correlations of 0.89 to 0.99 between the fitted and the observed values. Herein, the focus is on the graphical representations of the estimated contributions of impulses to responses (Figs. 5a, b, c) in the data-generating process (Fig. 2) operationalized with the three-equation model (Table S2). The contribution of each impulse to response was generated using the time series and break and regime impulse MGARCH(1,1) coefficients. The coefficients were used as weights applied to the appropriate observed time series and break and regime impulse coefficients, and then summed to obtain the total contribution (time series + breaks and regimes) of each impulse to each response.

According to the data-generating process (Fig. 2), the three equations included two foundational impulses: 1) deaths in the population 65 + in the CoM; and 2) the triage protocols. However, breaks and regimes in hospital referrals were not associated with the publication of the triage protocols in the univariate analyses, while a decrease in hospital referrals occurred before their publication, and an increase afterward. Therefore, the triage protocols were not considered in the MGARCH(1,1) model. However, LTCF resident hospital referrals began to decrease on Mar06-2020 (Fig. 4c,d), a day before centralized management of hospital beds was initiated and two days before LTCFs were locked down by the GCM (Table 2). The retreat of restrictions to hospital referrals was announced on Apr07-2020 (Table 2), six days after hospital referrals began to increase on Apr01-2020 (Fig. 4d). Therefore, the results of the three-equation MGARCH(1,1) model were compared in and after the period of restrictions to access to hospital care implemented by GCM.

### Contribution to hospital referrals

In Fig. 5a.1, the contribution of deaths in the population 65 + living in the community to hospital referrals (blue line) is compared with the estimated daily hospital referrals from the full MGARH(1,1) (broken black line) (See, Additional File 2 Table S2 Eq. 1B.1&2). Except for daily variation, the curves follow similar patterns though hospital referrals were overestimated in the GCM intervention period and underestimated in the interval between the end of the period of restrictions of hospital referral and the end of the March–April COVID-19 2020 epidemic (Fig. 5a.1). Also, deaths in the population 65 + in the CoM contributed to a lowering of hospital referrals in the GCM intervention period, while their contributions increased starting on Apr01-2020, 6 days after the publication of the last version of the triage protocol and 6 days before the retreat of the restrictions on hospital referrals by the GCM. In the pre and post-COVID-19 periods, the curves for the MGARCH(1,1) Eq. 1 and for deaths in the population 65 + were parallel and stable. Also, fewer daily hospital referrals occurred in the post than in the pre-COVID-19 period.

The negative contribution of deaths in the population 65 + living in the community to hospital referrals in the COVID-19 ascending period was not expected. Figure 5a.2 shows how it resulted (green line) from a conjunction of sources: 1. A positive contribution from the time series coefficients on deaths in the population 65 + (blue line); and 2. A strong negative contribution from breaks and regimes (red line) starting with the ascending period of the COVID-19 epidemic followed by a strong positive contribution starting at the peak of the epidemic on Apr01-2020.

### Contribution to in-LTCF deaths

Deaths in the population 65 + living in the community contributed to in-LTCF deaths (green line, Fig. 5b.1) leaving a large contribution to the lagged coefficients for in-LTCF deaths (Additional File 2, Table S2, Eq. 2A). This contribution is shown as the area between the green and the broken black line in Fig. 5b.1 (see also Table S2, Eq. 2 B1a&b).

Hospital referral total contribution (orange line) was too small to be assessed in Fig. 5b.1 (Table S2, Eq. 2 B2a&b). A change of scale (Fig. 5b.2) shows that they increased in the winter months of January and February 2020. They also increased in the GCM intervention period and felt afterward to increase in the post-COVID period at a higher level than in the pre-COVID-19 period. However, hospital referral total contribution to in-LTCF deaths varied in a narrow range of more or less 12 cases per day from January 5 to June 27.

The contributions of breaks and regimes to in-LTCF deaths were small and happened mostly outside of the COVID-19 epidemic and GCM intervention period (Additional File 2, Table S2, Eq. 2 B1b and B2b).

### Contribution to LTCF resident in-hospital deaths

Lagged coefficients for LTCF resident in-hospital deaths were not statistically significant (Table S2, Eq. 3 A). Thus, Fig. 5c.1 compared the contribution of deaths in the population 65 + living in the community (green line) with the observed LTCF resident in-hospital deaths (broken black line). While the contribution of deaths in the population 65 + living in the community increased at the beginning of the GCM intervention period, it folded down at the COVID-19 peak and decreased thereafter. The positive contribution of deaths in the population 65 + to LTCF resident in-hospital deaths preceded the decrease in observed in-hospital deaths producing a pattern of discrepancies through time between the two time series distributions (Fig. 5c.1). The discrepancies can be traced down to the contribution of hospital referrals and in-LTCF deaths to in-hospital deaths (Table S2, Eq. 3 B2&B3). In Fig. 5c.2, the sum of the time series, break and regime contributions of both impulses are graphed (see Additional File 3, Figures S3a,b, for the specific contributions of the time series and the break and regime contributions). The contributions of hospital referrals (red line) to LTCF in-hospital deaths fell with the introduction of the early GCM interventions and suddenly increased a few days before the retreat of the restrictions to hospital referrals by the GCM. In-LTCF deaths' negative contribution (red line) to LTCF resident in-hospital deaths began four to five days after the introduction of the early GCM interventions. The decrease stopped four to five days after the contribution of hospital referrals became positive. From then on, in-LTCF deaths contributed positively to in-hospital deaths, passing by their pre-COVID levels. In the pre and post-COVID-19 period, hospital referrals and in-LTCF death contributions to in-hospital deaths were on par (Fig. 5c.2).

### Volatility in responses and adjustment of estimated to observed responses

**Fig. 6:**
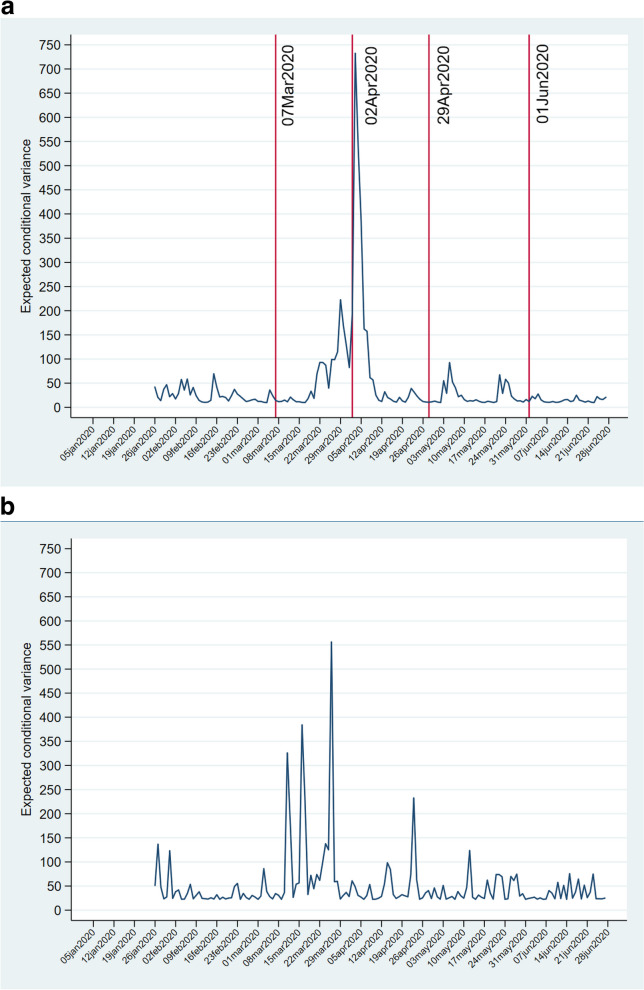
Conditional variances. **a** Daily in-LTCF deaths with breaks and regimes. **b** Daily in-hospital deaths

Volatility associated with the COVID-19 epidemic was obtained for the responses as estimated in the MGARCH(1,1) model. Estimated hospital referrals did not show statistically significant volatility. The pattern of volatility (Fig. 6a) associated with estimated in-LTCF deaths occurred on the same day as the peak in the number of in-LTCF deaths. Volatility of in-hospital deaths occurred over the whole Jan05/June27-2020 period with a peak on March 27, the day with the highest number of deaths in the population 65 + in the CoM (Fig. 6b).

**Fig. 7:**
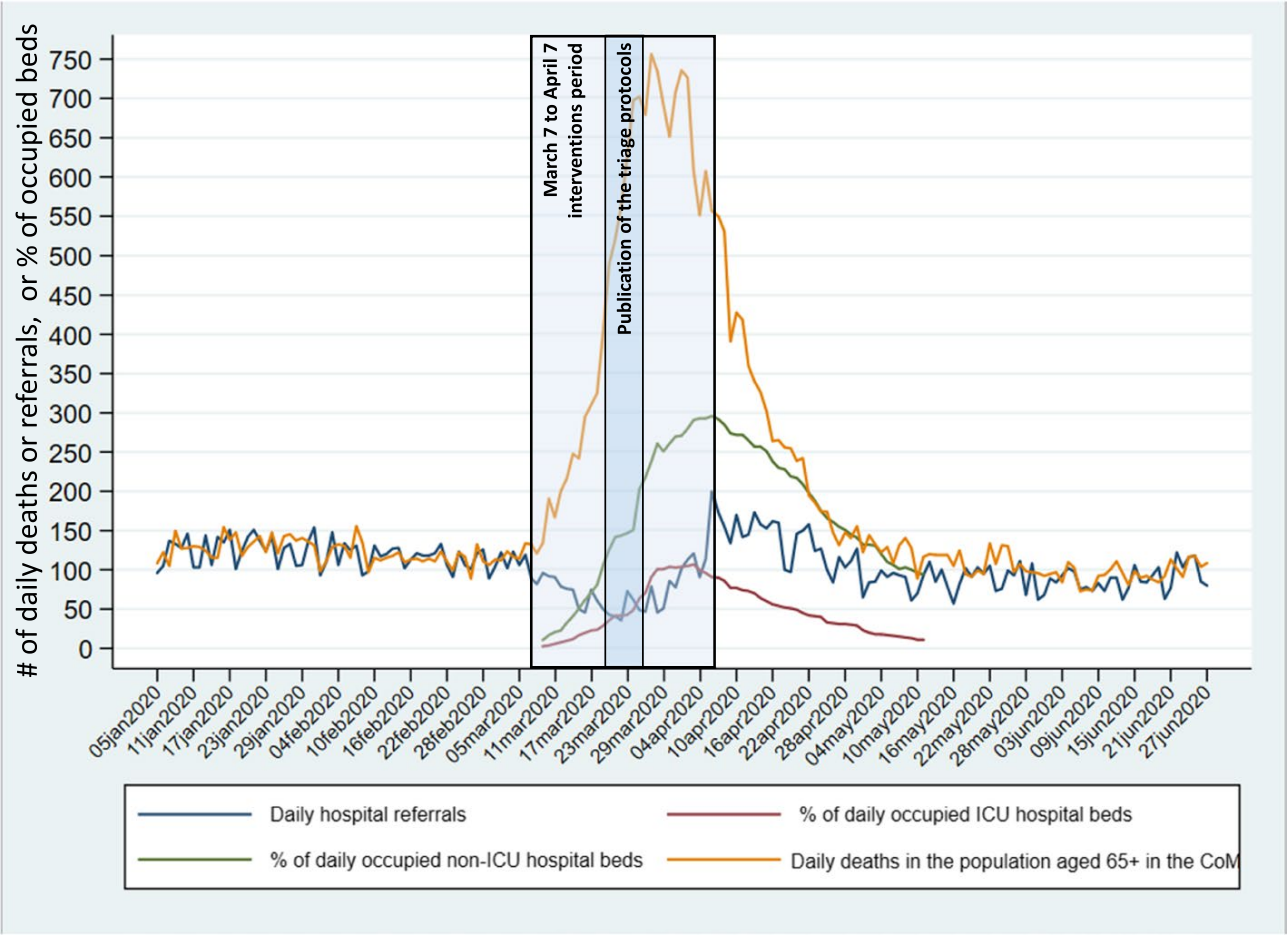
Hospital bed occupancy, deaths in the population aged 65 + , and hospital referrals of LTCF residents

Volatility in LTCF-resident in-hospital deaths happened in conjunction with the growing scarcity of hospital resources for non-COVID-19 patients in the Feb25/Apr28-2020 peak period of the COVID-19 epidemic (Figs. 6b and 7). Condes & Arribas [34] and Sanchez-Ubeda et al. [28] documented the overflow of ICU and non-ICU hospital beds at the end of March 2020. All acute care hospital beds in the CoM were occupied from Mar27-2020 to Apr5-2020 while the ICU bed occupancy rate was over 250% in the same period [28]. The peak of bed occupancy coincided with the Apr2-2020 peak of expected volatility for in-LTCF deaths. Meanwhile, COVID-19 patients occupied 90% of ICU beds and two-thirds of non-ICU beds [28]. Increases in deaths in the CoM population aged 65 + and decreases in hospital referrals of LTCF residents occurred in synchrony with the increasing occupancy of hospital ICU and non-ICU beds by COVID-19 patients (Fig. 7) and with the start of centralized management group through the GCM’s Ministry of Health’s *Gerencia de Hospitales’* (Hospital Management Group). After the enactment of the fourth version of the triage protocols on Mar25-2020, non-ICU, and ICU bed occupancy increased to their highest point from Mar27-2020 to Apr4-2020.

### Discussion

#### Summary of results

The main results of the analyses are summarized into the following seven points:

1. While hospital decrease of referrals from LTCFs were not associated with the publication dates of the triage protocols (Mar18/25–2020), they were associated with the GCM intervention period (Mar7/Apr7-2020).
2. In the GCM intervention period, the population 65 + in the CoM contributed to a decrease in hospital referrals and an increase after the publication of the triage protocols. After the partial retreat of the restrictions to access to hospital care, hospital referrals decreased with deaths in the population 65 + .
3. Deaths in the population 65 + living in the community were a contributor to in-LTCF deaths, though previous in-LTCF deaths contributed a large part of in-LTCF deaths. Hospital referrals contribution to in-LTCF deaths was small in COVID-19 epidemic.
4. The early positive contribution of deaths in the population 65 + in the community to LTCF resident in-hospital deaths was deflated by hospital referrals and in-LTCF deaths in the GCM intervention period. In-LTCF deaths contributed positively to LTCF resident in-hospital deaths after the retreat of restrictions on hospital care.
5. The GCM interventions were interwoven with the COVID-19 epidemic phases. Restrictions on hospital referrals were implemented in the ascending phase of the epidemic, while their retreat occurred a week after the beginning of the descending phase.
6. Evidence was obtained for high levels of uncertainty – volatility – in the flux and management of dying persons in the population 65 + living in the community or LTCF. Episodes of volatility in the LTCF resident in-hospital deaths time series were obtained over the pre, post and COVID-19 periods.
7. Deaths in the population 65 + in the CoM and in-LTCFs, and ICU and non-ICU bed occupancy, and episodes of turbulence in in-LTCF deaths occurred in conjunction.

#### Interpretation of results

In the data-generating process, deaths in the population 65 + in the CoM were hypothesized to increase the number of daily hospital referrals, in-LTCF, and LTCF resident in-hospital deaths in the early GCM intervention period. Thereafter, the triage protocols were hypothesized to decrease hospital referrals and LTCF resident in-hospital deaths and contribute to an increase in in-LTCF deaths, while the COVID-19 epidemic pursued its course. Thus, a break and a change of regime in hospital referrals, in LTCF resident in-hospital and in-LTCF deaths were expected with the implementation of the triage protocols. However, these expected breaks and regimes were not identified in this study.

During the Feb25/Apr28-2020 peak period of the COVID-19 epidemic, the CoM’s hospitals were confronted with bed saturation and, in some cases, near collapse due to high levels of non-ICU and ICU bed occupancy [28, 34]. In this context, the GCM introduced the triage protocols restricting hospital referrals of LTCF residents with moderate to high levels of disabilities and cognitive impairments [13, 14]. They were introduced three weeks after the GCM's first interventions limiting access to hospital care by LTCF residents, which was also the start of the COVID-19 epidemic. In this study, data on disability and cognitive impairments of LTCF residents referred to hospitals were not available. However, in studies conducted on COVID-19 LTCF residents in two Madrid hospitals after the publication of the triage protocols, those hospitalized had significantly less disability and cognitive decline than those who were not hospitalized [35, 36]

Daily hospital referrals were sharply curtailed on Mar6-2020 when the first GCM administrative directions aimed at managing access and delivery of hospital care to LTCF residents were implemented. This break introduced a regime coterminous with falls in the daily number of LTCF resident in-hospital deaths. This suggests that changes in hospital referral practices were implemented from Mar6-2020 to Mar17-2020, while medicalization of LTCF was not implemented [32]. Local LTCF managers were confronted with growing barriers to access to hospital care [28], and there was evidence of disorganization of delivery of care within LTCF [10, 31]. Nonetheless, hospital referrals started to grow at the end of March 2020 with the time series peak for deaths in the population 65 + in the CoM, and with an increase in LTCF resident in-hospital deaths. Again, this suggests that the degree of adherence to hospital referral protocols was relaxed a few days after the publication of their last version on Mar25-2020. Thus, changes in hospital referral practices and protocols occurred throughout the GCM intervention period. The publication of the triage protocols was one intervention among many.

The triage protocols were excluded from the multivariate analyses as none of the expected changes in LTCF hospital referrals and daily deaths occurred in conjunction with their publication dates. The multivariate analyses showed that hospital referral contributions to in-LTCF deaths were small. The main contributor to in-LTCF deaths was deaths in the population 65 + in the CoM, leaving a large role for previous in-LTCF to day-to-day in-LCTF deaths. This suggests a large contribution to in-LTCF deaths of COVID-19 contagion among LTCF residents and of factors associated with high levels of contagion in LTCFs [12, 13].

Lagged coefficients for LTCF resident in-hospital deaths were not statistically significant. Thus, there was no correlation through time between in-hospital daily deaths of LTCF residents. This result is in line with the lower level of volatility in LTCF in-hospital deaths than among in-LTCF deaths.

The positive contribution, in the ascending phase, of deaths in the population 65 + living in the community to LTCF resident in-hospital deaths was compensated by the negative contributions of hospital referrals and in-LTCF deaths. These negative contributions started with the GCM intervention period. The triage protocols did not modify this negative pace. The negative trends ended more or less with the peak of deaths in the population 65 + . Hospital referral and in-LTCF death contributions to LTCF resident in-hospital deaths turned positive before the retreat of the restriction to access to hospital care by the GCM.

As indicated by the results of the MGARCH(1,1) Eq. 2 on the lagged coefficients of in-LTCF deaths and volatility, LTCFs were hit early in the COVID-19 epidemic with the challenges of providing health and social care to severely ill and dying residents in times of high turbulence, restricted access to hospital care, and very limited access to in-LTCF care and alternatives to hospital care. Also, hospital referrals of LTCF residents were reduced in the ascending phase of COVID-19 when they were needed. Finally, hospital referrals and in-LTCF deaths were associated with reduced LTCF resident in-hospital deaths.

The data-generating process in Fig. 2 was based on two foundational impulses: the Feb25/Apr28-2020 period of the COVID-19 epidemic in the CoM and the Mar18/25–2020 triage protocols. This study focuses on protocols central to the public debate on CoM policies during the COVID-19 epidemic. The results of the study showed that the triage protocols were not associated with further decreases in hospital referrals or LTCF resident in-hospital deaths. Rather, hospital referrals began to decrease on March 6 coinciding with increasing daily deaths in the CoM population aged 65 + (Mar1-2020) and with increasing ICU and non-ICU hospital bed occupancy. These results suggest modifications to the data-generating process (Fig. 2):

1. The second foundational impulse, triage protocols, is deleted.
2. The availability of ICU and non-ICU hospital beds is introduced as the second foundational impulse.
3. The third foundational impulse is the set of interventions introduced by the GCM between Mar7/Apr7-2020. The GCM interventions changed the interplay between the impulses and responses and radically modified LTCF residents' access to hospital care.
4. Decreases in hospital referrals and increases in in-LTCF deaths in the GCM intervention period contributed to decreases in LTCF resident in-hospital deaths.
5. LTCFs were experiencing a largely locally generated COVID-19 contagion process.
6. Deaths in the Madrid aged population contribution to in-LTCF deaths suggests that LTCFs were not able to stop COVID-19 from invading their premises.
7. Hospitals were largely protected against turbulence attributable to hospital referrals of LTCF residents.
8. Turbulence associated with the daily deaths of LTCF residents in the Feb25/Apr28-2020 period of the COVID-19 epidemic was concentrated in LTCFs.

#### Limitations and strengths

There are no official statistics on the deaths of LTCF residents in Spain [37]. During the epidemic period, the CoM Ministry of Social Policies, Families, Equality and Fertility collected information on the deaths of LTCF residents by place of death. Thus, prediction equations to obtain the estimated pre-COVID-19 LTCF resident in-hospital daily deaths were modeled using a data-generating process (Fig. 2). Therefore, LTCF residents' daily in-hospital deaths during the pre-COVID-19 period were imputed based on hospital referrals, in-LTCF daily deaths, and the CoM population aged 65 + in the COVID-19 and post-COVID-19 periods. The imputation model was validated with the estimation of LTCF resident in-hospital deaths in the post-COVID-19 period.

Restrictions on hospital referrals of LTCF residents did not apply to those who had private health insurance [38]. An unknown number of LTCF residents with private health insurance during the epidemic had access to private ambulance services and referrals to private hospitals. However, the number of LTCF residents admitted to private hospitals in the Feb25/Apr28-2020 period of the COVID-19 epidemic is known to be small [39]. Finally, health policy restrictions were applied only to public hospitals, not to private hospitals [39]

This study has several strengths. The data-generating process (Fig. 2) focused on the objectives of the CoM triage protocols and their implementation in the context of the Feb25/Apr28-2020 period of the COVID-19 epidemic. The expected coefficients and their signs were hypothesized. Statistical tests on estimated coefficients examined the extent of their alignment with the data-generating process. Causality was not inferred from the results of the analyses.

The data are based on official statistics of hospital referrals and deaths and use advanced and appropriate statistical methods. All LTCF resident deaths between January 5, 2020, and June 27, 2020, were included in this study. Univariate statistical tests were used to identify breaks associated with the triage protocols. Before testing breaks, time series were examined. Breaks were tested on appropriately transformed time series. The multivariate analysis was based on a data-generating process. The MGARCH(1,1) procedure was selected based on the volatility of the univariate analyses. The results of the MGARCH(1.1) were reliable and based on tests on residuals and unit roots. Statistical tests and estimated MGARCH(1,1) coefficients obtained from the statistical methods used in this paper were interpreted using graphical representations.

### Conclusion

Six conclusions can be drawn from this research:

1. Results of the multivariate time series analyses provide an evidence-based description of the contribution of the GCM interventions to hospital referrals, in-LTCF and in-hospital LTCF residents' deaths in the pre-COVID, the ascending and descending phases of the COVID-19, and in the post-COVID periods in the CoM.
2. Decreases in LTCF residents' hospital referrals and LTCF resident in-hospital deaths preceded the enactment of the triage protocol by two weeks. Thus, the data-generating process, driven by the objectives and means of the triage protocols, was rejected. A new data-generating process was proposed.
3. The GCM interventions were associated opposite to the phases of the COVID-19 epidemic. In the first week of the ascending phase, the GCM interventions blocked hospital referrals leading to a decrease in LTCF resident in-hospital deaths. Trends in hospital referrals and LTCF resident in-hospital deaths showed that GCM interventions were relaxed with the beginning of the descending phase of the COVID-19 epidemic.
4. Two impulses ought to be included in a modified data-generating process in analyses provided data can be retrieved from official archives: a) hospital ICU and non-ICU bed occupancy; and b) GCM policy interventions, other than the triage protocols, restricting hospital care for LTCF residents.
5. The Mar18/25–2020 triage protocols were implemented as one of the GCM interventions in the COVID-19 first wave. Starting on Mar7-2020, the GCM interventions were already pursuing and implementing the objectives of the triage protocols restricting LTCF residents’ referrals to hospitals, while the number of LTCF residents dying in hospitals decreased.
6. LTCFs were hit by a strong locally generated COVID-19 contagion process, while the medicalization of LTCFs planned by the GCM was not implemented.
7. The situation of the CoM’s LTCFs was the epitome of point 21 of the European Parliament resolution on the COVID-19 pandemic [37], which stressed that EU member states focused on preserving hospital capacity while neglecting to provide needed care to LTCF residents, resulting in excessive mortality.

### Supplementary Information

Supplementary Material 1

Supplementary Material 2

Supplementary Material 3

## Data Availability

The datasets used and/or analyzed during the current study are available from the corresponding author on reasonable request.

## References

[1] Sapiano MRP, Dudeck MA, Soe M, Edwards JR, O’Leary EN, Wu H, et al. Impact of coronavirus disease 2019 (COVID-19) on US Hospitals and Patients, April-July 2020. Infect Control Hosp Epidemiol. 2022;43:32–9. 33602380 10.1017/ice.2021.69 PMC7943952

[2] Mateen BA, Wilde H, Dennis JM, Duncan A, Thomas N, McGovern A, et al. Hospital bed capacity and usage across secondary healthcare providers in England during the first wave of the COVID-19 pandemic: A descriptive analysis. BMJ Open. 2021;11:e042945. 33500288 10.1136/bmjopen-2020-042945 PMC7843315

[3] Verelst F, Kuylen E, Beutels P. Indications for healthcare surge capacity in European countries facing an exponential increase in coronavirus disease (COVID-19) cases, March 2020. Eurosurveillance. 2020;25:2000323. 32265003 10.2807/1560-7917.ES.2020.25.13.2000323 PMC7140594

[4] Sepulveda ER, Stall NM, Sinha SK. A Comparison of COVID-19 Mortality Rates Among Long-Term Care Residents in 12 OECD Countries. J Am Med Dir Assoc. 2020;21:1572-1574.e3 33138940 10.1016/j.jamda.2020.08.039 PMC7486852

[5] Aalto UL, Pitkälä KH, Andersen-Ranberg K, Bonin-Guillaume S, Cruz-Jentoft AJ, Eriksdotter M, et al. COVID-19 pandemic and mortality in nursing homes across USA and Europe up to October 2021. Eur Geriatr Med. 2022;13:705–9. 35299261 10.1007/s41999-022-00637-1 PMC8929245

[6] Islam N, García López FJ, Jdanov DA, Royo-Bordonada MA, Khunti K, Lacey B, et al. Unequal impact of the Covid-19 pandemic on excess deaths, life expectancy, and premature mortality across Spanish regions in 2020 and 2021. Gaceta Sanitaria 2024 (in press). 10.1016/j.gaceta.2024.102424 39500260

[7] Martinez-Peromingo J, Serra-Rexach JA. Long-Term Care Facilities and the COVID-19 Pandemic: Lessons Learned in Madrid. J American Geriatric Society. 2020;68:1920–2. 10.1111/jgs.16665 PMC7323397 32557547

[8] Zunzunegui MV, Rico M, Béland F, García-López FJ. The Impact of Long-Term Care Home Ownership and Administration Type on All-Cause Mortality from March to April 2020 in Madrid. Spain Epidemiologia. 2022;3:323–36. 36417241 10.3390/epidemiologia3030025 PMC9620910

[9] Koleva G, Rico M, García López FJ, Figuera D, Padilla J, García M. The impact of COVID-19 in nursing homes in Madrid, Spain: a need for assessment. Lancet Reg Health Eur. 2021;11:100261. 10.1016/j.lanepe.2021.100261. 10.1016/j.lanepe.2021.100261 PMC8566164 34751264

[10] Zalakaín J, Davey V. Suárez-González A. The COVID-19 on users of long-term care services in Spain. LTCcovid, International Long-Term Care Policy Network, CPEC-LSE, 28/05/2020 https://ltccovid.org/wp-content/uploads/2020/05/LTCcovid-Spain-country-report-28-May-1.pdf

[11] Comas-Herrera A, Zalakaín J, Lemmon E, Henderson D, Litwin C, Hsu AT, et al. Mortality associated with COVID-19 in care homes: international evidence. 2020. https://ltccovid.org/wp-content/uploads/2021/02/LTC_COVID_19_international_report_January-1-February-1-2.pdf

[12] Zunzunegui MV, Béland F, Rico M, López FJG. Long-Term Care Home Size Association with COVID-19 Infection and Mortality in Catalonia in March and April 2020. Epidemiologia. 2022;3:369–90. 36417245 10.3390/epidemiologia3030029 PMC9620903

[13] Zunzunegui MV, Béland F, García López FJ. Restrictions on Hospital Referrals from Long-Term Care Homes in Madrid and COVID-19 Mortality from March to June 2020: A Systematic Review of Studies Conducted in Spain. Epidemiologia. 2023;4:176–87. 37367184 10.3390/epidemiologia4020019 PMC10296840

[14] Reyero ZA. Morirán de forma indigna. Madrid: K.O. ediciones; 2022.

[15] Rico M. Los seis documentos que demuestran que Ayuso miente sobre la orden de no trasladar enfermos de residencias a hospitales. 2020. https://www.infolibre.es/politica/seis-documentos-demuestran-ayuso-miente-orden-no-trasladar-enfermos-residencias-hospitales_1_1183785.html. Accessed 31 July 2024.

[16] Dirección General de Coordinación Sociosanitaria. Consejería de Salud. Comunidad de Madrid. Protocolo de coordinación - Madrid -Covid- 25/03/2020. 2020;:1–7. https://amyts.es/wp-content/uploads/2020/01/COVID-CAM-coordinaci%C3%B3n-residencias-sinfecha-20200412.pdf. Accessed 19 Oct 2023.

[17] Dirección General de Coordinación Sociosanitaria. Consejería de Sanidad de la Comunidad de Madrid. Protocolo de coordinación - Madrid - Covid - 18/03/2020. 2020;:1–5. https://files.mediaset.es/file/10002/2020/05/19/PROTOCOLO_DE_COORD-_PARA_LA_ATENCION_DE_PACIENTES-PDF_-2_2b93.pdf. Accessed 6 Apr 2024.

[18] Government of the Community of Madrid. Memoria 2020. Servicio Madrileño de Salud. 2020. https://gestiona3.madrid.org/bvirtual/BVCM050404.pdf. Accessed 6 Apr 2024.

[19] Zhang XS, Charland K, Quach C, Nguyen QD, Zinszer K. Institutional, therapeutic, and individual factors associated with 30-day mortality after COVID-19 diagnosis in Canadian long-term care facilities. J Am Geriatr Soc. 2022. 10.1111/jgs.17975. 35906882 10.1111/jgs.17975 PMC9353371

[20] Costa-Font J, Jiménez Martin S, Viola A. Fatal Underfunding? Explaining COVID-19 Mortality in Spanish Nursing Homes. J Aging Health. 2021;33:607–17. 33818164 10.1177/08982643211003794 PMC8236671

[21] Joan Costa-Font & Sergi Jiménez & Cristina Vilaplana Prieto & Analía Viola, 2022. "Long-term Care in Spain," Studies on the Spanish Economy eee2022–23, FEDEA.

[22] Consejo de MInistros. Plan de desescalada. 2020. https://www.lamoncloa.gob.es/consejodeministros/Paginas/enlaces/280420-enlace-desescalada.aspx. Accessed 6 Apr 2024.

[23] Ditzen J, Karavias Y, Westerlund J. Testing and estimating structural breaks in time series and panel data in Stata. Discussion Papers. 2021. p. 21-14. https://ideas.repec.org/p/bir/birmec/21-14.html. Accessed 31 July 2024.

[24] Rapach DE, Strauss JK. Structural breaks and GARCH models of exchange rate volatility. J Appl Economet. 2008;23:65–90. 10.1002/jae.976

[25] Schaffer AL, Dobbins TA, Pearson SA. Interrupted time series analysis using autoregressive integrated moving average (ARIMA) models: a guide for evaluating large-scale health interventions. BMC Med Res Methodol. 2021;21. 10.1186/s12874-021-01235-8 PMC7986567 33752604

[26] Becketti S. Introduction to Time Series Using Stata. Revised edition. 2020.

[27] Stata. StataCorp. Stata Statistical Software: Release 15. College Station, TX: StataCorp LLC; 2017.

[28] Sánchez-Úbeda EF, Sánchez-Martín P, Torrego-Ellacuría M, Del Rey-Mejías A, Morales-Contreras MF, Puerta JL. Flexibility and bed margins of the Community of Madrid's hospitals during the first wave of the SARS-CoV-2 pandemic. Int J Environ Res Public Health. 2021;18:3510. 10.3390/ijerph18073510. 10.3390/ijerph18073510 PMC8036372 33800638

[29] European Parliament resolution on the COVID-19 pandemic: lessons learned and recommendations for the future. 12/07/2023. https://www.europarl.europa.eu/doceo/document/TA-9-2023-0282_EN.html.

[30] Tribunal Superior de Justicia de Madrid. Auto 55/2020, de 6 de mayo de 2020. Sala de lo Contencioso-Administrativo. Caso Ayuntamiento de Leganés. 2020. https://www.idhuv.es/wp-content/uploads/2023/10/autoTSJM_Leganes_6mayo2020.pdf. Accessed 31 July 2024.

[31] Amnesty International. Abandonadas a su suerte. La desprotección y discriminación de las personas mayores en residencias durante la pandemia COVID-19 en España. 2020. https://www.amnesty.org/fr/wp-content/uploads/sites/4/2022/04/EUR4155022020SPANISH.pdf. Accessed 31 July 2024.

[32] Supremo T, Auto 10862, 2023, Sala de lo Contencioso-Administrativo, de 20 de julio de,. Caso Ayuntamiento de Leganés. Procedimiento. 2023;7770(2022):2023.

[33] Galaup L, Caballero F. Cronología del desastre en las residencias de Madrid: 7.600 fallecidos, crisis en el Gobierno e investigación judicial. ElDiario.es. 2020. https://www.eldiario.es/madrid/cronologia-residencias-madrid-judicializada-gobierno_1_6064645.html. Accessed 31 July 2024.

[34] Condes E, Arribas JR. Impact of COVID-19 on Madrid hospital system. Enferm Infecc Microbiol Clin. 2021;39:256–7. 10.1016/j.eimc.2020.06.005 PMC7315960 38620683

[35] García-Cabrera L, Pérez-Abascal N, Montero-Errasquín B, Rexach Cano L, Mateos-Nozal J, Cruz-Jentoft A. Characteristics, hospital referrals and 60-day mortality of older patients living in nursing homes with COVID-19 assessed by a liaison geriatric team during the first wave: a research article. BMC Geriatr. 2021;21:610. 10.1186/s12877-021-02565-4. 10.1186/s12877-021-02565-4 PMC8553905 34715807

[36] Bielza R, Sanz J, Zambrana F, Arias E, Malmierca E, Portillo L, et al. Clinical Characteristics, Frailty, and Mortality of Residents With COVID-19 in Nursing Homes of a Region of Madrid. J Am Med Dir Assoc. 2021;22:245-252.e2. 33417840 10.1016/j.jamda.2020.12.003 PMC7833075

[37] Zunzunegui MV, García López FJ, Rodríguez V. Unknown mortality of the population in long-term care homes in Spain. Gac Sanit 2023;37:102261. 10.1016/j.gaceta.2022.102261. 10.1016/j.gaceta.2022.102261 36308997

[38] Peinado F. Los mayores con seguro privado sí fueron trasladados de residencias a hospitales en Madrid. El País. 2020. https://elpais.com/espana/madrid/2020-06-10/los-mayores-con-seguro-privado-pudieron-ser-trasladados-de-residencias-a-hospitales-en-madrid.html. Accessed 31 July 2024.

[39] Peinado F. Un empresario de hospitales y otro de residencias desmontan la defensa de Ayuso en la crisis de los geriátricos. El País. 2020. https://elpais.com/espana/madrid/2020-10-09/un-empresario-de-hospitales-y-otro-de-residencias-desmontan-la-defensa-de-ayuso-en-la-crisis-de-los-geriatricos.html. Accessed 31 July 2024.

