# Supplementary material for: Hospital referrals, exclusions from hospital care, and deaths among long-term care residents in the Community of Madrid during the March–April 2020 COVID-19 epidemic period: a multivariate time series analysis
Source: BMC Geriatr. 2024 Aug 14;24:682. doi: 10.1186/s12877-024-05254-0 (PMC11323618; doi:10.1186/s12877-024-05254-0)
Supplement: Supplementary file 1 — Supplementary Material 1 [file 12877_2024_5254_MOESM1_ESM.docx]

**Hospital referrals, exclusions from hospital care,**

**and deaths among long-term care residents in the Community of Madrid**

**during the March-April 2020 COVID-19 epidemic period:**

**A multivariate time series analysis**

**Additional File 1**

**François Béland**

**Maria Victoria Zunzunegui**

**Fernando J. García López**

[**Francisco Pozo-Rodriguez**](https://pubmed.ncbi.nlm.nih.gov/?term=Pozo-Rodriguez+F&cauthor_id=29370849)

**March 27, 2024**

**Additional File 1**

Before testing for breaks, time series were examined for six properties: stationarity, volatility, residuals white noise, unit roots of residuals, and the number of autocorrelations (AC) and moving average (MA) terms. Four steps were used to examine the properties of total daily deaths in the Community of Madrid population aged 65+ and of deaths of persons living in the community, in-LTCF (Long-Term Care Facilities) and in-hospital deaths of LTCF residents, and daily hospital referrals:

1. AR and MA terms introduced in ARIMA were examined with plots of autocorrelations (AC) and partial autocorrelations (PAC) (Figures not shown – available on request).
2. Stationarity was tested first with Phillips & Perron's (PP) unit-root tests, and with the Dickey-Fuller (DF) test [1]. The number of lag terms was estimated with the DF-generalized least-square (DFGLS) test [1]. Second, if the unit root tests were not rejected, the Lee-Strazicich (LS) unit-root tests [2, 3] with two breaks were run. If the LS unit-root tests were not rejected, then day-to-day changes – that is first differences – in time series were used in the analysis.
3. ARIMA models were tested. The number of AR and MA terms suggested by AC, PAC plots, and DF-GLS tests were considered. Observed daily events or first differences were considered as indicated by the results of the tests on stationarity. Log-likelihood ratio tests (LRT) were used to select AR and MA terms entering the ARIMA models (Table not shown);
4. GARCH(1,1) models were set up using results from the ARIMA models, and *arch* and *garch* parameters were examined for volatility in the time series [4].

Results of tests for stationarity, volatility, the white noise of residuals, and unit root tests on residuals are shown in Table S1.

The autocorrelations collapsed toward zero at lags ten to eleven. The partial autocorrelation graphs showed a dominant term in all but the hospital referrals time series (Figures not shown – available on request). PP unit root tests were run with the number of appropriate lags for each time series. The time series for daily deaths in the CoM population aged 65+ and for daily in-LTCF resident deaths were not stationary while the time series for deaths of persons living in the community, daily in-hospital deaths and hospital referrals for LTCF residents were stationary on the PP unit root tests (Table S1). The DF tests for stationarity could not be rejected on four of the five time series. Therefore, the LS unit-root tests with two breaks were run on the four series. The LS unit-root tests rejected the null hypothesis of unit roots.

ARIMA models were tested on each of the time series. White noise tests on residuals were significant except for references to hospitals. Thus, ARIMA models were run *de novo* on the first differences. White noise tests for residuals were not rejected (Table S1). First differences were used in the break tests for the variables, except for references to hospitals.

AR and MA terms for each time series obtained from the ARIMA models were included in the GARCH(1,1) models. Tests for *arch* and/or *garch* terms were significant on the five time series.

| **Table S1. Unit root and volatility tests on univariate time series** | | | |
| --- | --- | --- | --- |
| **and white noise and unit root tests on residuals** | | | |
|  |  | |  |
| **A. Unit root tests** | **Phillips & Perron's** | **Augmented** | **Lee-Strazicich** |
|  | **unit root test** | **Dickey-Fuller**  **unit root tests** | **unit root tests** |
|  |  |  | **with two breaks** |
|  | **P-value** | **P-value** | **P-value** |
| **a. Deaths - Madrid aged 65+** | Not rejected@0.35 | Not rejected@0.43 | Rejected@0.01 |
| **b. Deaths - Living in the community** | Rejected@0.01 | Rejected@>0.001 | -- |
| **c. Deaths in residence - LTCH residents** | Not rejected@0.34 | Not rejected@0.31 | [Rejected@0.01](mailto:Rejected@0.01) |
| **d. Deaths in hospital - LTCH residents** | [Rejected@0.01](mailto:Rejected@0.01) | Not rejected@0.09 | [Rejected@0.01](mailto:Rejected@0.01) |
| **e. Referrals to hospitals -LTCH residents** | [Rejected@0.01](mailto:Rejected@0.01) | Not rejected[@0.052](mailto:Rejected@0.05) | Rejected@0.05 |
|  |  |  |  |
| **B. Volatility** | **Arch terms** | **Garch terms** | **Arch + Garch** |
|  | **[Coef. & P-value]** | **[Coef. & P-value]** |  |
|  |  |  |  |
| **a. Deaths - Madrid aged 65+** | 0.137;0.012 | 0.800>0.001 | 0.937 |
| **b. Deaths - Living in the community** | 0.213;0.009 | 0.692>0.001 | 0.905 |
| **c. Deaths in residence - LTCH residents** | 0.236;0.002 | 0.763;>0.001 | 0.999 |
| **d. Deaths in hospital - LTCH residents** | 0.242;0.059 | 0.713; >0.001 | 0.955 |
| **e. Referrals to hospital - LTCH residents** | 0.315;0.025 | Not significant | -- |
|  |  |  |  |
| **C. Tests on residuals** | **White noise test** | | **Phillips & Perron's** |
|  | **P-value for** | **P_value for** | **Unit root test** |
|  | **Portmanteau's Q** | **Bartlett's B** | **for residuals** |
|  |  |  | **P-value** |
| **a. Deaths - Madrid aged 65+** | 0.706 | 0.369 | >0.001 |
| **b. Deaths - Living in the community** | 0.033 | 0.805 | >0.001 |
| **b. Deaths in residence - LTCH residents** | 0.289 | 0.140 | >0.001 |
| **c. Deaths in hospital - LTCH residents** | 0.078 | 0.592 | >0.001 |
| **d. Referrals to hospitals -LTCH residents** | 0.617 | 0.223 | >0.001 |

Only the *arch* term was significant with hospital referrals. The sums of the *arch* and *garch* terms were less than one (Table S1). Volatilities were low in pre-COVID-19 and post-COVID-19 periods (Figures S1a.1, S1a.2, b, c, and d). However, though the COVID-19 epidemic started on Feb25-2020, volatilities remained on the low side until Mar17/23-2020, as if increasing daily deaths were not associated with volatility. The disruptions in the time series occurred with or after the publication of the triage protocols.

Volatilities for daily deaths in the CoM population 65+ (total and for those living in the community) reached high levels at the peak of the COVID-19 epidemic (Figures S1a.1, S1a.2).

- **Figure S1. Statistically significant volatility in the impulses and responses:**
- **Conditional variance in univariate Garch equations**

-
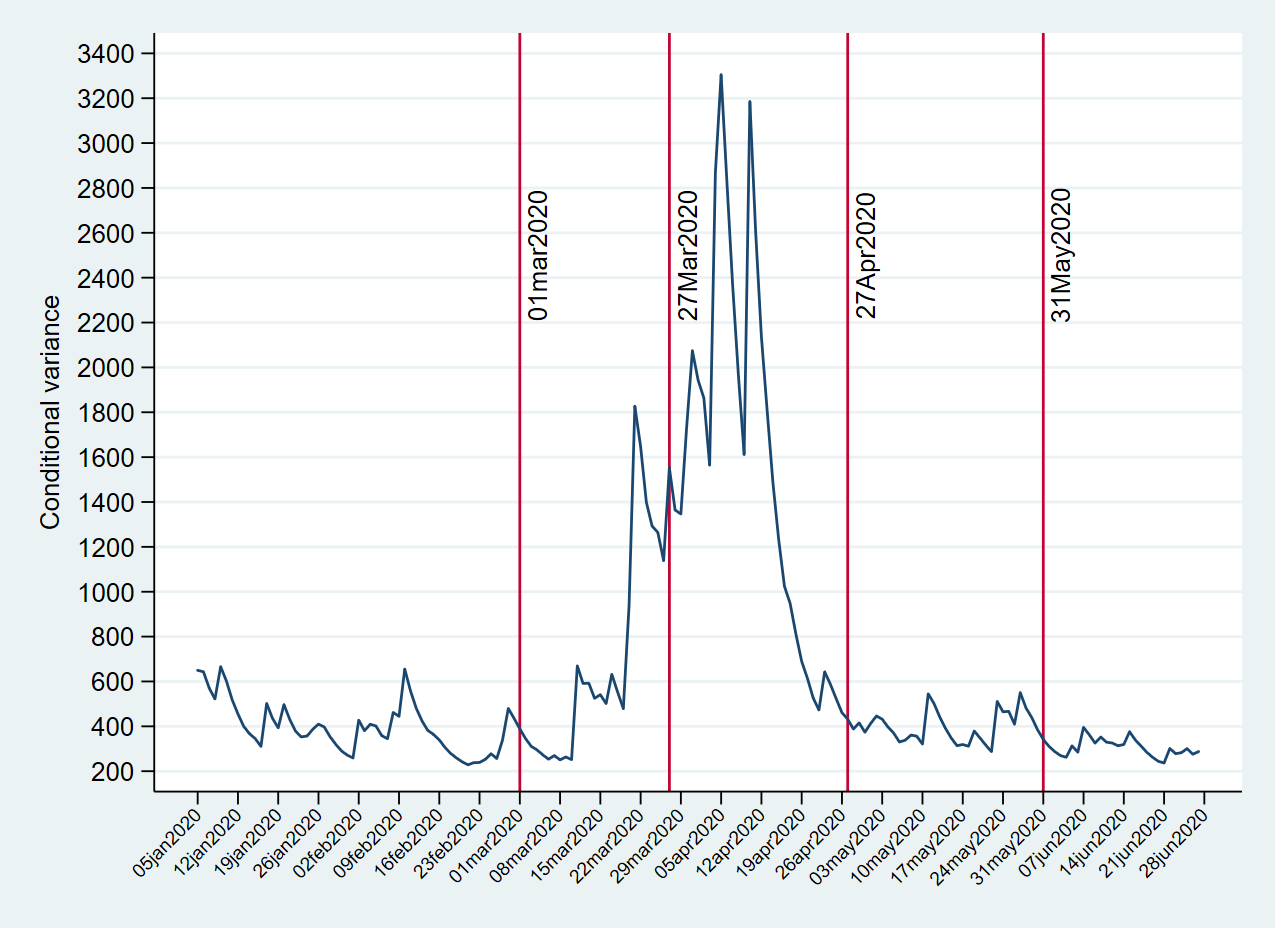


**Publication of the triage protocols**

FIG. S1a.1: Conditional variance: daily deaths in the population 65+ in the CoM


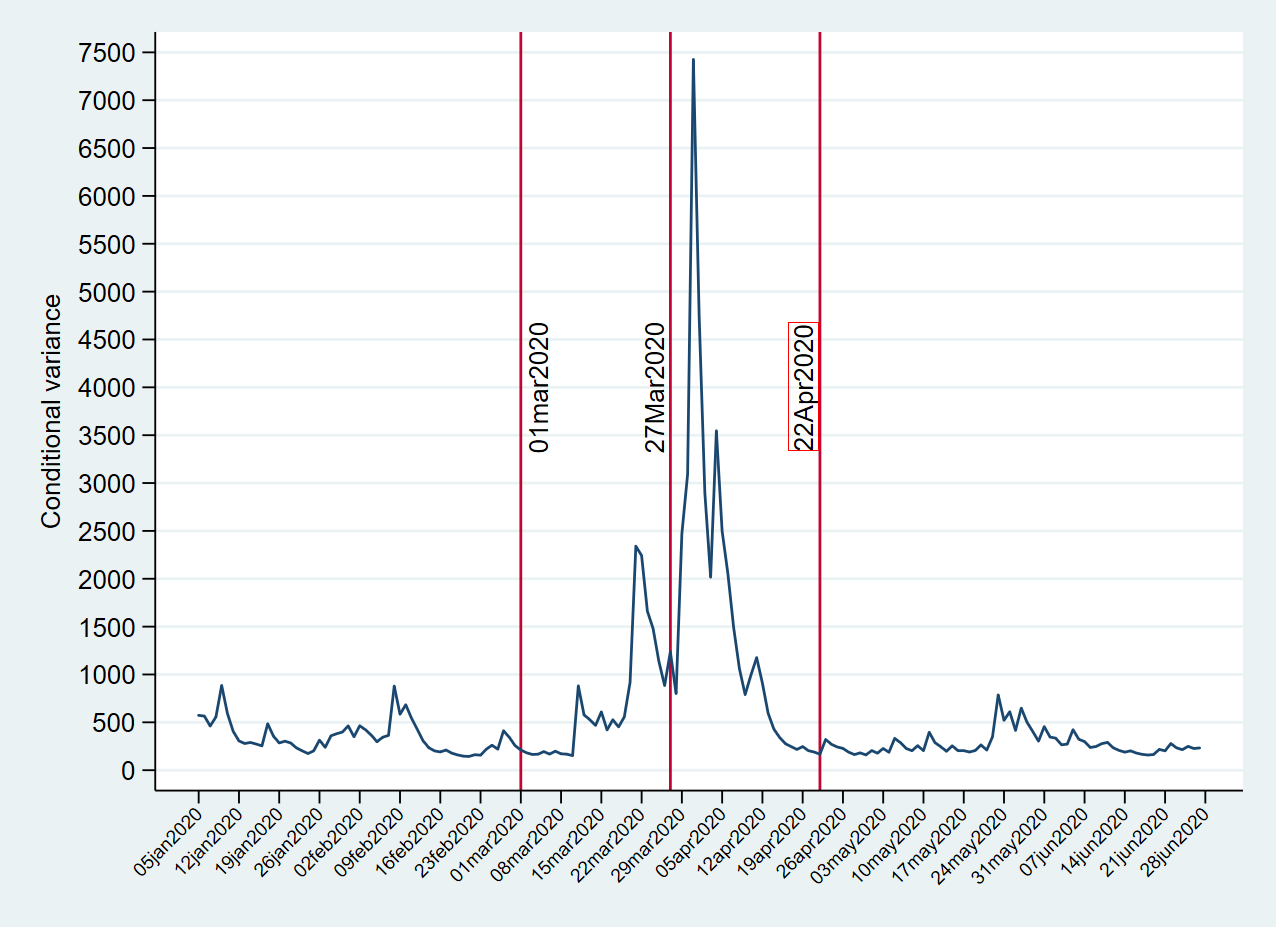


**Publication of the triage protocols**

FIG. S1a.2: Conditional variance: daily deaths in population 65+ in the CoM

living in the community


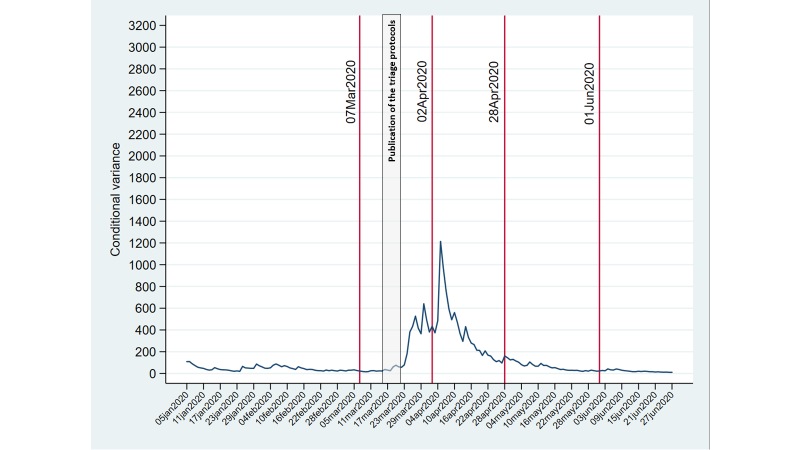


FIG S1b: Conditional variance: daily in-LTCF deaths


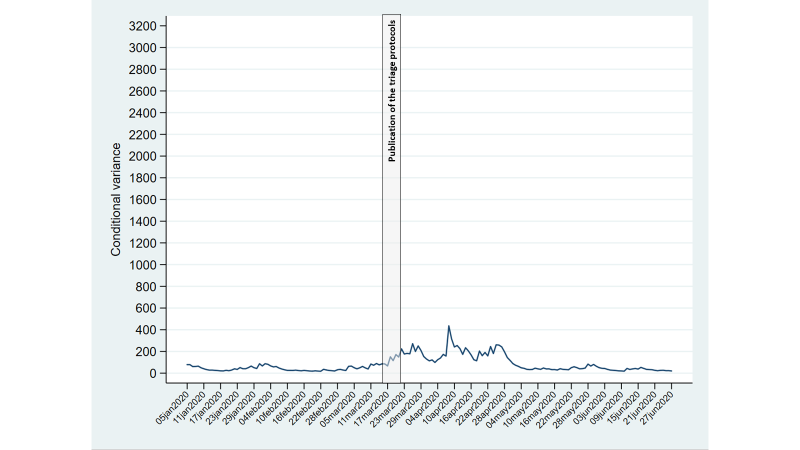


FIG S1c: Conditional variance: daily in-hospital deaths of LTCF residents


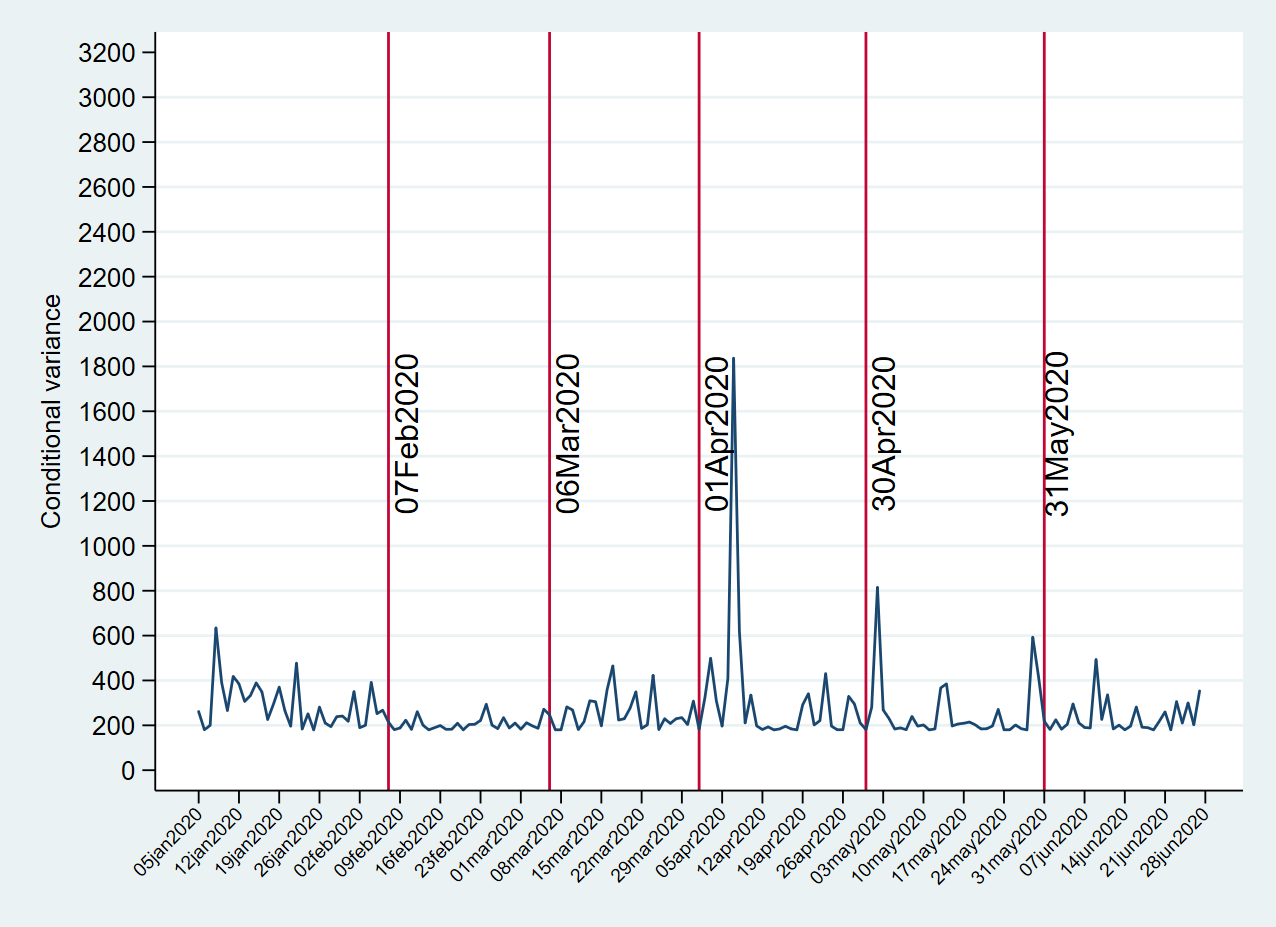


FIG S1d: Conditional variance: daily hospital referrals of LTCF residents

LTCF resident hospital referrals and in-LTCF and in-hospital deaths peaked at the end of the GCM interventions on Apr7-2020 (Figures S1b, c). On Apr28-2020, volatilities returned to their pre-COVID period. Volatilities in daily hospital referrals and LTCF resident in-LTCH and in-hospital deaths were small compared with volatility in the population 65+ in the CoM. Volatility in hospital referrals showed a pattern of small peaks from January 5 to June 27, 2020 (Figure S1c). These results are indicative of a state of lower-level disruptions in the hospital-based time series and a high level in the population 65+ and in-LTCF deaths.

**References**

1. Enders W., Applied Econometric Time Series, Wiley, 2004

2. Jewell T, Lee J, Tieslau M, Strazicich MC. Stationarity of health expenditures and GDP: Evidence from panel unit root tests with heterogeneous structural breaks. J Health Econ. 2003;22:313–23.

3. Rapach DE, Strauss JK. Structural breaks and GARCH models of exchange rate volatility. Journal of Applied Econometrics. 2008;23:65–90.

4. Ditzen J, Karavias Y, Westerlund J. Testing and Estimating Structural Breaks in Time Series and Panel Data in Stata. 2021.
