# Supplementary material for: Hospital referrals, exclusions from hospital care, and deaths among long-term care residents in the Community of Madrid during the March–April 2020 COVID-19 epidemic period: a multivariate time series analysis
Source: BMC Geriatr. 2024 Aug 14;24:682. doi: 10.1186/s12877-024-05254-0 (PMC11323618; doi:10.1186/s12877-024-05254-0)
Supplement: Supplementary file 2 — Supplementary Material 2 [file 12877_2024_5254_MOESM2_ESM.docx]

**Hospital referrals, exclusions from hospital care,**

**and deaths among long-term care residents in the Community of Madrid**

**during the March-April 2020 COVID-19 epidemic period:**

**A multivariate time series analysis**

**Additional File 2**

**François Béland**

**Maria Victoria Zunzunegui**

**Fernando J. García López**

[**Francisco Pozo-Rodriguez**](https://pubmed.ncbi.nlm.nih.gov/?term=Pozo-Rodriguez+F&cauthor_id=29370849)

**June 18, 2024Additional File 2**

Based on the results of the unit root, volatility, and white noise tests (Additional File 1), the MGARCH(1,1) model was chosen for the multivariate analysis. Residuals of the MGARCH(1,1) models were tested for white noise with Bartlett's periodogram [33] and Portmanteau's [33] tests. Additionally, residuals were examined for unit roots with Phillips & Peron's tests [33] (Table S2).

Hospital referrals, deaths in LTCFs, and deaths in hospitals of LTCF residents were responses in the data-generating model (Figure 2). Three steps were used to examine the contribution of impulses to responses in the MGARCH(1,1). In the first step, for each equation in Table S2, the time series and break and regime impulse coefficients were tested for statistical significance (CI @ 0.95) in a GARCH(1,1) model. Second, for each equation, statistically significant coefficients on all impulses were entered simultaneously in a GARCH(1,1) model, and retained if statistically significant. Third, all significant coefficients were entered in a MGARCH(1,1) model, and retained if statistically significant.

The final results of the MGARCH(1.1) analysis are reported in Table S2.

*Adjustment of estimated to observed response time series and volatility*

Observed and estimated daily hospital referrals are plotted in Figure S2a. The estimated time series followed the general shape of the observed time series. Divergences were limited to small day-to-day variations. Hospital referrals were well predicted by daily deaths in the population aged 65 and over.

*Arch* and *garch* terms for volatility were not statistically significant for daily hospital referrals in the MGARCH(1,1) model. The null hypothesis of white noise in the residuals was not rejected (Table S2, Equation 1, Section D)). Phillips & Perron’s test [1] rejected the null hypothesis of a unit root in the residuals (Table S2, Equation 1, Section E).

Observed and predicted values of daily deaths in LTCFs are plotted in Figure S2b. The two distributions showed few discrepancies, while daily variations were small. Volatilities for daily death in LTCFs were statistically significant (Table S2, Equation 2, section C). They were flat in the pre and post-COVID-19 periods, while peaked during the COVID-19 epidemic (Figure 6a), indicating uncertainties and disturbances in daily deaths in LTCFs.

Daily deaths in hospitals of LTCF residents were reproduced by the model (Figure S2c). More estimated, than observed, in-hospital deaths were obtained in the first week of the COVID-19 epidemic. Volatilities were statistically significant for daily deaths in hospitals of LTCF residents (Table S2, Equation 3, Section C). Small increases of volatility in the pre and post-COVID-19 periods were obtained suggesting a seasonal pattern not associated with the COVID-19 epidemic. In the COVID-19 period, volatilities occurred as surges on specific days. White-Noise and unit root tests behaved as expected (Table S2, Equation 3, Sections D, E).

|  | **Table S2. MGARCH multivariate model** | | |  |  |
| --- | --- | --- | --- | --- | --- |
| **for hospital referrals, deaths within residences, and in hospitals** | | | | |  |
|  |  |  |  |  |  |
|  | **Coefficients** | **Standard** | **P-levels** | **95% C.I.** | |
|  |  | **Errors** |  |  |  |

| ***Equation 1: Daily hospital referrals*** |  |  |  |  |  |
| --- | --- | --- | --- | --- | --- |
|  |  |  |  |  |  |
| **A) Response: Previous daily hospital referrals** | |  |  |  |  |
| Lag(7) | 0.432 | 0.063 | 0.000 | 0.309 | 0.554 |
| Lag(12) | -0.239 | 0.053 | 0.000 | -0.343 | -0.136 |
| **B) Impulse: Daily deaths in the population 65+ in the CoM** | |  |  |  |  |
| **1. Time series coefficients** |  |  |  |  |  |
| Lag(5) | 0.088 | 0.041 | 0.031 | 0.008 | 0.167 |
| Lag(8) | -0.079 | 0.044 | 0.068 | -0.165 | 0.006 |
| Lag(10) | 0.130 | 0.043 | 0.003 | 0.046 | 0.215 |
| Lag(14) | 0.125 | 0.041 | 0.002 | 0.045 | 0.206 |
| Lag(16) | -0.105 | 0.035 | 0.003 | -0.173 | -0.036 |
| **2. Breaks and regime** |  |  |  |  |  |
| **Shift:** |  |  |  |  |  |
| Mar27-2021 | 36.268 | 16.499 | 0.028 | 3.931 | 68.605 |
| **Pulses:** |  |  |  |  |  |
| Jan5/Feb29-2020 | 22.514 | 3.887 | 0.000 | 14.895 | 30.133 |
| Mar01/Mar26-2020 | 33.561 | 6.672 | 0.000 | 20.483 | 46.638 |
| Mar27/Apr26-2020 | -85.436 | 22.599 | 0.000 | -129.729 | -41.143 |
| **Ramps:** |  |  |  |  |  |
| Mar01/Mar26-2020 | -3.973 | 0.556 | 0.000 | -5.062 | -2.884 |
| Mar27/Apr26-2020 | 2.998 | 0.802 | 0.000 | 1.426 | 4.571 |
| **3. Constant** | 1.481 | 3.182 | 0.642 | -4.755 | 7.718 |
| **C) Volatility** |  |  |  |  |  |
| Constant | 182.725 | 20.616 | 0.000 | 142.317 | 223.132 |
| **D) White-Noise tests** |  |  |  |  |  |
| Bartlett's B | 0.680 |  | 0.740 |  |  |
| Portmanteau's Q | 47.950 |  | 0.182 |  |  |
| **E) Unit Root P&P test** |  |  | > 0.000 |  |  |

| ***Equation 2: In-LTCF daily deaths*** |  |  |  |  |  |
| --- | --- | --- | --- | --- | --- |
|  |  |  |  |  |  |
| **A) Response: Previous in-LTCF daily deaths** |  |  |  |  |  |
| Lag(1) | 0.151 | 0.069 | 0.029 | 0.016 | 0.286 |
| Lag(2) | 0.146 | 0.073 | 0.045 | 0.003 | 0.288 |
| Lag(6) | 0.152 | 0.063 | 0.016 | 0.028 | 0.276 |
| Lag(9) | 0.13 | 0.056 | 0.021 | 0.020 | 0.24 |
| Lag(10) | -0.209 | 0.048 | 0.000 | -0.303 | -0.115 |
| **B) Impulses:** |  |  |  |  |  |
| **1. Daily deaths in the population 65+ in the CoM living in the community** | | | |  |  |
| **a. Time series coefficients** |  |  |  |  |  |
| Lag(0) | 0.071 | 0.024 | 0.003 | 0.024 | 0.116 |
| Lag(1) | 0.064 | 0.023 | 0.004 | 0.02 | 0.110 |
| Lag(3) | 0.114 | 0.027 | 0.000 | 0.062 | 0.167 |
| Lag(6) | 0.080 | 0.027 | 0.003 | 0.027 | 0.133 |
| Lag(8) | 0.078 | 0.027 | 0.004 | 0.025 | 0.130 |
| Lag(11) | 0.060 | 0.026 | 0.020 | 0.009 | 0.110 |
| Lag(12) | 0.069 | 0.028 | 0.014 | 0.014 | 0.124 |
| **b. Breaks and regime** |  |  |  |  |  |
| **Pulse:** |  |  |  |  |  |
| Jan05/Feb29-2020 | -10.141 | 4.002 | 0.011 | -17.984 | -2.299 |
| Apr27/May30-2020 | -3.522 | 1.653 | 0.033 | -6.762 | -0.281 |
| **Ramps:** |  |  |  |  |  |
| Jan05/Feb29-2020 | 0.168 | 0.073 | 0.013 | 0.025 | 0.31 |
| **2. Hospital referrals** |  |  |  |  |  |
| **a. Time series coefficients** |  |  |  |  |  |
| Lag(3) | -0.056 | 0.027 | 0.035 | -0.109 | -0.004 |
| Lag(4) | 0.058 | 0.026 | 0.026 | 0.007 | 0.110 |
| Lag(6) | -0.062 | 0.024 | 0.009 | -0.109 | -0.015 |
| **b. Breaks and regime** |  |  |  |  |  |
| **Ramp:** |  |  |  |  |  |
| Apr01/27Jun-2020 | -0.130 | 0.049 | 0.008 | -0.269 | -0.034 |
| **3. Constant** | 1.814 | 0.964 | 0.060 | -0.074 | 3.702 |
| **C) Volatility** |  |  |  |  |  |
| Constant | 1.555 | 1.151 | 0.177 | -0.701 | 3.812 |
| Arch(1) | 0.233 | 0.079 | 0.003 | 0.077 | 0.388 |
| Garch(1) | 0.729 | 0.082 | 0.000 | 0.567 | 0.890 |
| **D) White-Noise tests** |  |  |  |  |  |
| Bartlett's B | 0.95 |  | 0.322 |  |  |
| Portmanteau's Q | 50.484 |  | 0.124 |  |  |
| **E) Unit Root P&P test** |  |  | > 0.000 |  |  |

| ***Equation 3: In-hospital daily deaths*** |  |  |  |  |  |
| --- | --- | --- | --- | --- | --- |
|  |  |  |  |  |  |
| **A)     Response: Previous in-hospital deaths** | n.s. | n.s. | n.s. | n.s. | n.s. |
| **B)      Impulses:** |  |  |  |  |  |
| **1. Daily deaths in the population 65+ in the CoM** | |  |  |  |  |
| **a. Lagged time series coefficients** |  |  |  |  |  |
| Lag(2) | 0.146 | 0.017 | 0.000 | 0.113 | 0.180 |
| Lag(15) | 0.091 | 0.021 | 0.000 | 0.049 | 0.133 |
| **2. Hospital referrals** |  |  |  |  |  |
| **a. Time series coefficients** |  |  |  |  |  |
| Lag(4) | -0.109 | 0.023 | 0.000 | -0.154 | -0.064 |
| Lag(7) | 0.054 | 0.027 | 0.045 | 0.001 | 0.107 |
| Lag(14) | 0.044 | 0.021 | 0.034 | 0.003 | 0.085 |
| **b. Breaks and regime** |  |  |  |  |  |
| **Ramp :** |  |  |  |  |  |
| Mar05/Mar31-2020 | -1.099 | 0.186 | 0.000 | -1.463 | -0.734 |
| **3. In-LTCF daily deaths** |  |  |  |  |  |
| **a.  Time series coefficients** |  |  |  |  |  |
| Lag(3) | -0.094 | 0.023 | 0.000 | -0.140 | -0.048 |
| Lag(12) | 0.141 | 0.021 | 0.000 | 0.100 | 0.183 |
| **b. Breaks and regime** |  |  |  |  |  |
| **Pulse :** |  |  |  |  |  |
| Apr01/28-2020 | -14.461 | 5.030 | 0.004 | -24.319 | -4.603 |
| **Ramp :** |  |  |  |  |  |
| Apr01/28-2020 | 0.792 | 0.228 | 0.001 | 0.344 | 1.239 |
| **4. Constant** | 1.688 | 0.794 | 0.033 | 0.133 | 3.244 |
| **C) Volatility** |  |  |  |  |  |
| Constant | 0.802 | 0.226 | 0.000 | 0.359 | 1.246 |
| Arch(1) | 0.802 | 0.226 | 0.000 | 0.359 | 1.246 |
| **D) White-Noise tests** |  |  |  |  |  |
| Bartlett's B | 0.750 |  | 0.620 |  |  |
| Portmanteau's Q | 49.134 |  | 0.153 |  |  |
| **E) Unit Root P&P test** |  |  | > 0.000 |  |  |
|  |  |  |  |  |  |
| ***Correlations between predicted time series*** |  |  |  |  |  |
|  |  |  |  |  |  |
| **Equations 1 and 2** | 0.245 | 0.079 | 0.002 | 0.090 | 0.400 |
| **Equations 1 and 3** | 0.015 | 0.087 | 0.861 | -0.155 | 0.185 |
| **Equations 2 and 3** | -0.091 | 0.082 | 0.266 | -0.252 | 0.070 |

- **Figure S2.1 Adjustment of estimated response time series to observed time series**


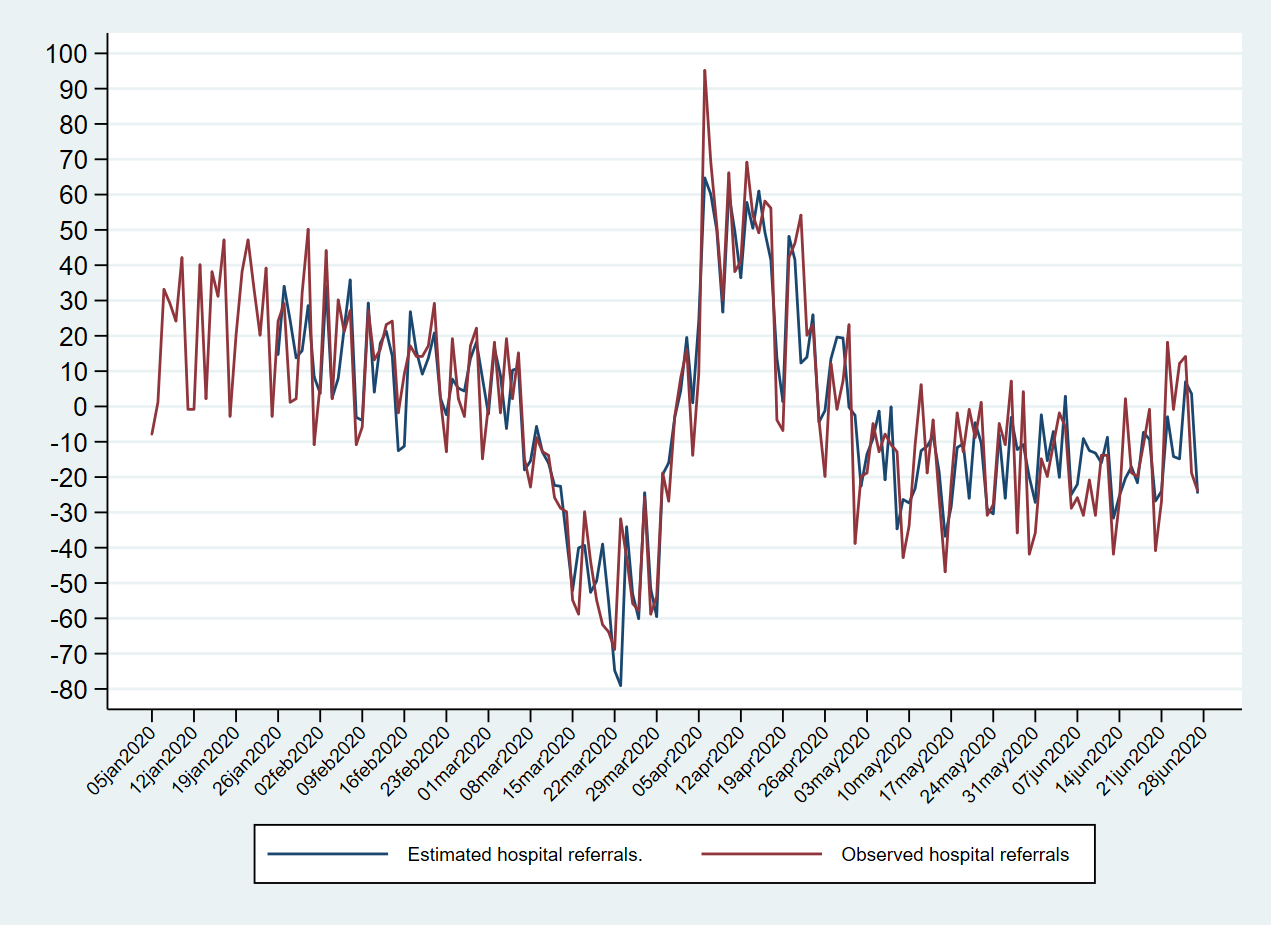


- FIG S2a. Observed and estimated number of daily hospital referrals


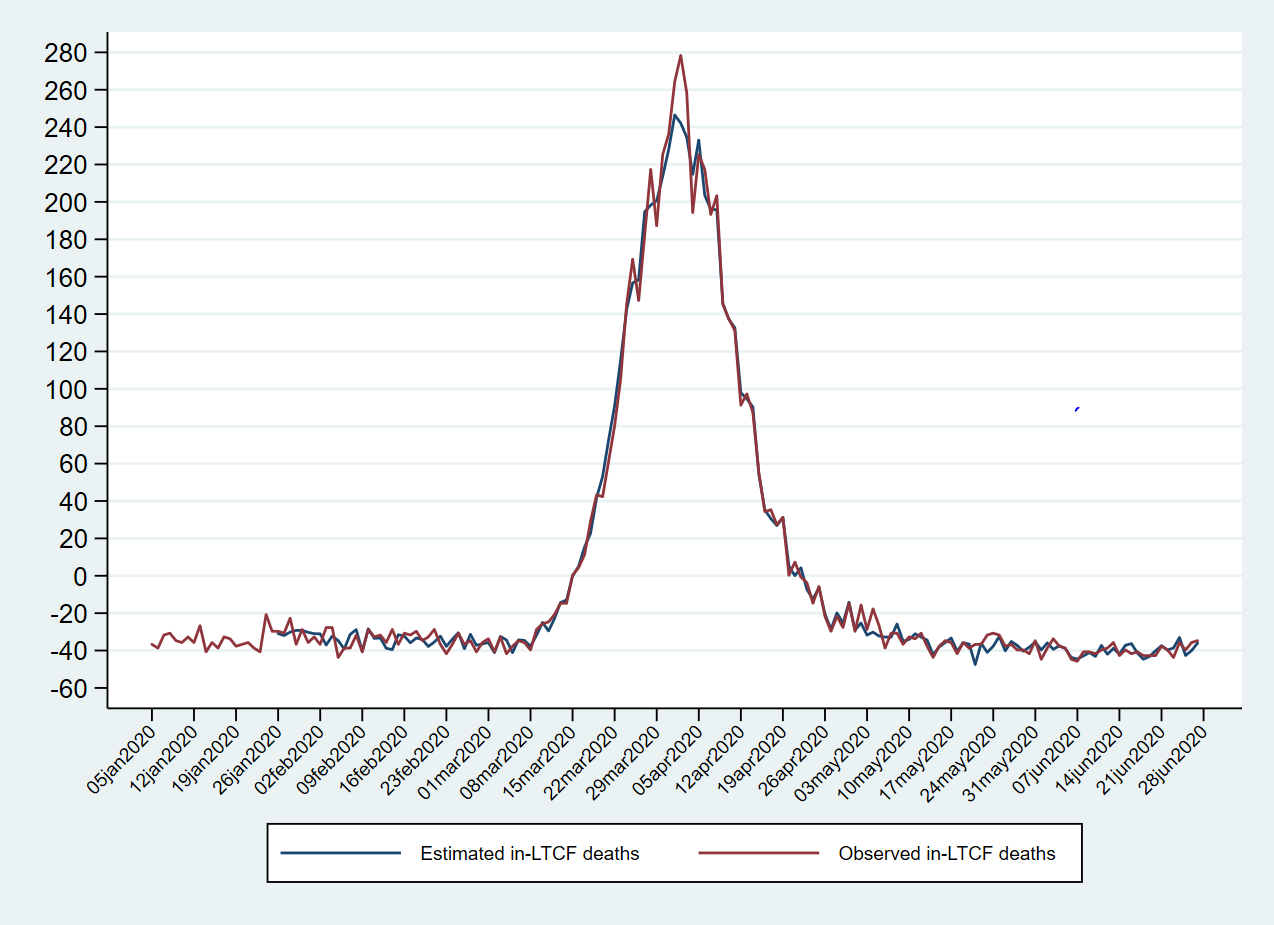


FIG S2b. Observed and estimated number of in-LTCF daily deaths


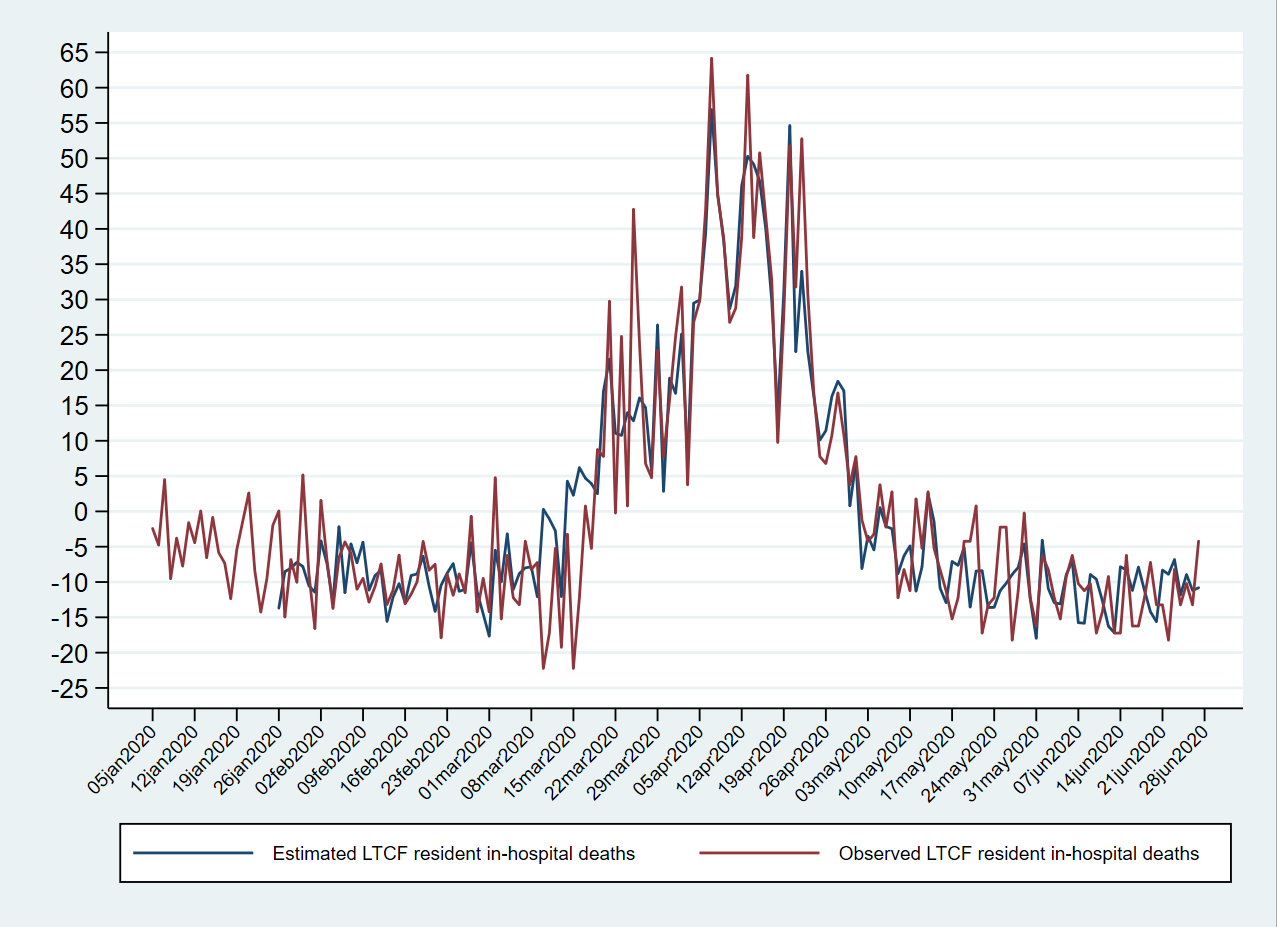


FIG S2c Observed and estimated numbers of LTCF residents’ in-hospital daily deaths

**Reference**

1. Phillips P.C.B., Perron P., Unit root in time series_Cowles_1988. Biometrika. 1988;75:335–46.
2. Stata. Stata Press. 2017.
