# Supplementary material for: Hospital referrals, exclusions from hospital care, and deaths among long-term care residents in the Community of Madrid during the March–April 2020 COVID-19 epidemic period: a multivariate time series analysis
Source: BMC Geriatr. 2024 Aug 14;24:682. doi: 10.1186/s12877-024-05254-0 (PMC11323618; doi:10.1186/s12877-024-05254-0)
Supplement: Supplementary file 3 — Supplementary Material 3 [file 12877_2024_5254_MOESM3_ESM.docx]

**Hospital referrals, exclusions from hospital care,**

**and deaths among long-term care residents in the Community of Madrid**

**during the March-April 2020 COVID-19 epidemic period:**

**A multivariate time series analysis**

**Additional File 3**

**François Béland**

**Maria Victoria Zunzunegui**

**Fernando J. García López**

[**Francisco Pozo-Rodriguez**](https://pubmed.ncbi.nlm.nih.gov/?term=Pozo-Rodriguez+F&cauthor_id=29370849)

**June 18, 2024Additional File 3**

**Figure S3 Contribution of impulses to responses in the MGARCH Equation 3**
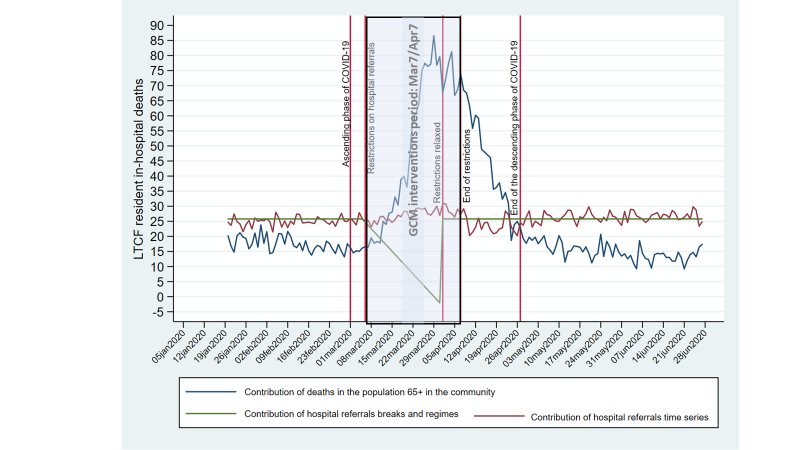


- FIG S3a. Contribution of hospital referrals and deaths in the population 65+ living in the community to LTCF residents’ in-hospital deaths
-
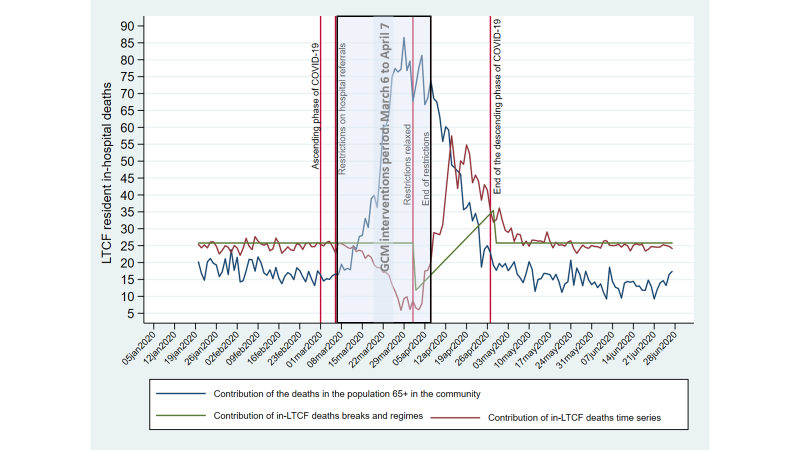

- FIG S3b. Contribution of in-LTCF deaths and deaths in the population 65+ living in the community to LTCF residents’ in-hospital deaths to hospital referrals
